# CD79a promotes CNS-infiltration and leukemia engraftment in pediatric B-cell precursor acute lymphoblastic leukemia

**DOI:** 10.1038/s42003-020-01591-z

**Published:** 2021-01-15

**Authors:** Lennart Lenk, Michela Carlet, Fotini Vogiatzi, Lea Spory, Dorothee Winterberg, Antony Cousins, Michaela Vossen-Gajcy, Olta Ibruli, Christian Vokuhl, Gunnar Cario, Omar El Ayoubi, Lisa Kramer, Matthias Ritgen, Monika Brüggemann, Robert Häsler, Martin Schrappe, Stephan Fuhrmann, Christina Halsey, Irmela Jeremias, Elias Hobeika, Hassan Jumaa, Ameera Alsadeq, Denis M. Schewe

**Affiliations:** 1 Department of Pediatrics I, ALL-BFM Study Group, Christian-Albrechts University Kiel and University Medical Center Schleswig-Holstein, Arnold-Heller-Str. 3, Haus C, 24105 Kiel, Germany; 2 Research Unit Apoptosis in Hematopoietic Stem Cells, Helmholtz Zentrum München, German Center for Environmental Health (HMGU), Marchioninistraße 25, 81377 Munich, Germany; 3 Institute of Cancer Sciences, College of Medical, Veterinary and Life Sciences, University of Glasgow, Garscube Estate, Switchback Road, Bearsden, Glasgow, G61 1QH UK; 4 Department of Pathology, Section of Pediatric Pathology, Venusberg-Campus 1, Gebäude 62, 53127 Bonn, Germany; 5 Institute of Immunology, Ulm University Medical Center, Albert-Einstein-Allee 11, 89081 Ulm, Germany; 6 Department of Medicine II, University Hospital Schleswig-Holstein, Langer Segen 8-10, 24105 Kiel, Germany; 7 Institute of Clinical Molecular Biology, Christian-Albrechts University Kiel and University Medical Center Schleswig-Holstein, Campus Kiel, Rosalind-Franklin-Straße 12, 24105 Kiel, Germany; 8 Department of Hematology and Oncology, HELIOS Hospital Berlin-Buch, Rosalind-Franklin-Straße 12, 24105 Kiel, Germany; 9 German Cancer Consortium (DKTK), Partnering Site Munich, Pettenkoferstr. 8a, 80336 München, Germany; 10 Department of Pediatrics, Dr. von Hauner Children’s Hospital, University Hospital, LMU Munich, Lindwurmstraße 4, 80337 München, Germany

**Keywords:** Preclinical research, Diagnostic markers, Acute lymphocytic leukaemia, Experimental models of disease

## Abstract

Central nervous system (CNS) involvement remains a challenge in the diagnosis and treatment of acute lymphoblastic leukemia (ALL). In this study, we identify CD79a (also known as Igα), a signaling component of the preB cell receptor (preBCR), to be associated with CNS-infiltration and –relapse in B-cell precursor (BCP)-ALL patients. Furthermore, we show that downregulation of CD79a hampers the engraftment of leukemia cells in different murine xenograft models, particularly in the CNS.

## Abstract (web summary)

Lenk et al find that the preB cell receptor (preBCR) is associated with infiltration and relapse of acute lymphoblastic leukemia in the central nervous system (CNS). They also show that downregulation of preBCR component CD79a reduces the engraftment of leukemia cells in different murine xenograft models, particularly in the CNS.

## Introduction

CNS-involvement is routinely assessed by microscopy of the cerebrospinal fluid (CSF)^1^, an approach that is limited in performance and informative value. Irrespective of the initial CNS status, all patients are treated with intrathecal chemotherapy which can be neurotoxic^2^. Hence, besides conclusive diagnostic markers, novel targets for specific eradication of leukemia cells in the CNS have to be established. In B-cell precursor (BCP)-ALL, certain cytogenetic alterations such as the t(1;19) translocation leading to the E2A-PBX1 fusion and the t(9;22) translocation causing the BCR-ABL fusion (Philadelphia-chromosome) are associated with a higher incidence of CNS-leukemia^1,3,4^. Recently we have reported that ZAP70 and the Interleukin-7 receptor (IL7R) are associated with CNS-involvement in BCP-ALL^5,6^. Both proteins are closely associated with the preBCR signaling pathway. The preBCR is composed of the heavy chain (µ), the surrogate light chain (VpreB and λ5) and the signaling components CD79a (Igα) and CD79b (Igβ)^7^. Activation of the preBCR results in the phosphorylation of tyrosine residues within the immunoreceptor tyrosine-based activation motifs (ITAMs) of the cytoplasmic tails of CD79a/CD79b. This is followed by subsequent recruitment and activation of Src homology kinases, such as LYN and FYN as well as spleen tyrosine kinase (SYK) and ZAP70^8^. Evidence suggesting that the preBCR is involved in the pathogenesis of lymphoid malignancies is accumulating^9^. Hence, we hypothesized that the preBCR-signaling complex itself is important for CNS-infiltration.

## Results and discussion

### CD79a has independent prognostic relevance for CNS-involvement and CNS-relapse in BCP-ALL patients

**Fig. 1:**
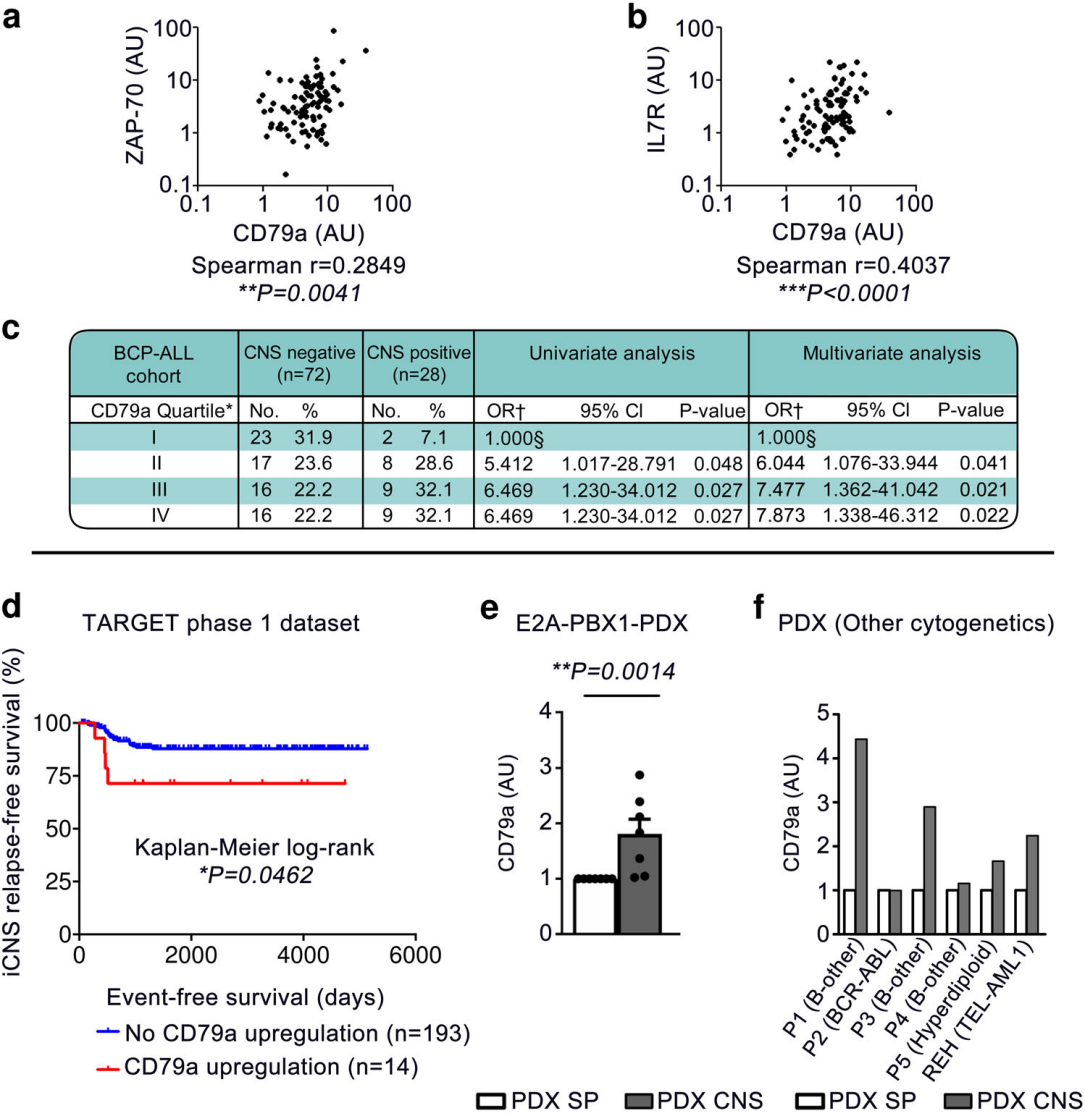
CD79a has independent prognostic relevance for CNS-involvement and CNS-relapse in BCP-ALL patients. **a**–**c** CD79a mRNA levels and further genes (all normalized to mRNA levels in the 697 cell line) were measured in diagnostic BM samples in a selected cohort of 100 pediatric BCP-ALL patients of mixed cytogenetics which contained 28 CNS-positive patients matched to 72 CNS-negative patients of corresponding sex and age^5^. **a**, **b** Bivariate correlation analysis between CD79a expression and **a** ZAP70 and **b** Interleukin-7-receptor (IL7R) were performed, Spearman correlation (two-tailed, respective 95% confidence interval (CI) [0.08789, 0.4605] and [0.2174, 0.5616]). **c** Univariate and multivariate logistic regression analysis for risk of initial CNS-involvement, controlled for age and white blood cell (WBC) count at diagnosis as well as TEL-AML and BCR-ABL positivity. ^*^Based on expression as measured by RT-PCR of patient material at initial diagnosis. ^†^Multivariate OR controlled for age and WBC count at diagnosis as well as TEL-AML and BCR-ABL positivity. ^§^Reference category. Definitions of patient CNS status in Supplementary Material **d** Kaplan–Meier survival curve showing reduced isolated CNS-relapse-free survival in children with upregulated CD79a gene expression in diagnostic BM/peripheral blood (upregulation is defined as *z*-score for gene expression ≥1.2 which is equivalent to the 11.5% top CD79a-expressing patients; TARGET phase 1 dataset). **e** E2A-PBX1 + BCP-ALL blasts from seven different patient-derived xenograft (PDX) samples were injected into NSG mice ALL cells were recovered from spleen (SP) and CNS and subjected to quantitative real-time PCR (qPCR). QPCR shows the upregulation of CD79a at the transcription level in PDX cells recovered from the CNS relative to cells isolated from Sp, Mann–Whitney-*U* test, two-tailed, graphs show mean with standard error of *n* = 7 independent samples. **f** An upregulation of CD79a was also observed in CNS-BCP-ALL cells compared to SP-BCP-ALL cells in NSG-mice injected with bone marrow cells from BCP-ALL patients of different cytogenetic backgrounds (*n* = 5 independent patient samples) or CNS-tropic REH cells (TEL-AML1) as determined via qPCR. Relative quantification of CD79a mRNA levels normalized to 697 cells are shown for individual patients, *P* Patient, *AU* Arbitrary units. *P < 0.05, **P ≤ 0.01, ***P ≤ 0.001.

To investigate this, we first measured CD79a mRNA levels in diagnostic bone marrow (BM) samples (70–99.5% blasts), in a cohort of 100 pediatric BCP-ALL patients^5^. This cohort was selectively designed to contain a high number of CNS-positive patients as only 3–5% of patients are CNS-positive upon initial diagnosis and CNS involvement is thus a rare event^1,10^. We found that CD79a levels significantly correlated with ZAP70 and with IL7R mRNA levels in bivariate correlation analyses (Fig. 1a, b). Furthermore, we found that patients diagnosed as CNS-positive showed significantly higher levels of CD79a than patients diagnosed as CNS-negative (median CD79a expression: 5.183 ± 0.396 in CNS− vs. 7.537 ± 1.278 in CNS+; Supplementary Fig. 1a, logarithmic scale). Importantly, multivariate logistic regression analysis, excluding the effect of known parameters associated with CNS-infiltration, showed that a CD79a expression above the 75th percentile was associated with a significant ~8-fold increased risk for CNS-positivity compared to the lower quartile (odds ratio = 7.873, 95% confidence interval (CI) [1.338,46.312], *p* = 0.022, Fig. 1c). CD79a expression levels above the 25th and 50th percentiles were also linked with CNS-involvement (Fig. 1c). Multivariate logistic regression analysis comparing the lower quartile to the second, third and fourth quartiles in CD79a expression revealed a significant 7-fold increased risk for CNS-positivity for patients with CD79a expression levels above the 25th percentile (odds ratio = 7.0, 95% CI [1.4,33.9], *p* = 0.016, Supplementary Fig. 1b) suggesting that CD79a up-regulation increases the risk for CNS-positivity.

Next, we investigated the association between CD79a and CNS-relapse in an independent cohort from the TARGET phase 1 data set containing gene expression data of more than 200 BCP-ALL cases. We found that an increased expression of CD79a (*z*-score ≥ 1.2) was associated with significantly reduced long-term probability rates for isolated-CNS-relapse-free survival (Fig. 1d). When analyzing all relapse cases associated with the CNS (combined and isolated), we found that high CD79 levels are associated with a tendency towards reduced CNS-relapse-free-survival (Supplementary Fig. 1c). We found no significant difference for BM-relapse-free probability rates between CD79a high and CD79a low patients further suggesting that CD79a is particularly important for CNS-relapse (Supplementary Fig. 1d).

We then asked if ALL cells retrieved from the CNS show elevated CD79a expression levels. For this approach we xenotransplanted NOD.Cg-*Prkdc*^*scid*^*Il2rg*^tm1Wjl^/SzJ (NSG) with diagnostic BM samples of seven different E2A-PBX^+^ patients (Supplementary Table 1, patients 1–7) and recovered PDX-ALL cells from different organs.

In line with previous reports^11^, PDX animals exposed some degree of meningeal engraftment, supporting the view that most ALL cells are in principle capable of invading the CNS. Indeed, we found a significant upregulation of CD79a in ALL cells from the CNS as compared to spleen PDX cells (Fig. 1e). Moreover, we found increased levels of CD79a in CNS-ALL cells compared to SP-ALL cells in 4/5 mice transplanted with PDX samples of further BCP-ALL subtypes (3×“B-other”, 1×BCR-ABL^+^, 1×hyperdiploid, Supplementary Table 1, patients 8–12) as well as in a mouse transplanted with the highly CNS-tropic TEL-AML1^+^ cell line REH (Fig. 1f).

Taken together, our data show that high expression levels of CD79a are associated with CNS-involvement in BCP-ALL patients of different cytogenetics indicating that CD79a, in conjunction with other markers, could be used as surrogate marker for the assessment of CNS-involvement in BCP-ALL. Furthermore, our data imply that CD79a is upregulated in ALL cells from the CNS in BCP-ALL xenograft models. Moreover, we observed that CD79a mRNA levels strongly correlate with mRNA levels of CD79b in our BCP-ALL patient cohort (Supplementary Fig. 1e) and that CD79b is upregulated in ALL cells recovered from the CNS versus SP from NSG-mice bearing E2A-PBX1-PDX cells (Supplementary Table 1, patients 1–7, Supplementary Fig. 1f) indicating that CD79b is also important for CNS involvement in BCP-ALL.

### CD79a is required for leukemic engraftment particularly in the CNS

Previous reports suggest that some BCP-ALL subtypes, e.g. E2A-PBX1^+^ BCP-ALL critically depend on preBCR signaling, and that others, including BCR-ABL^+^ BCP-ALL, progress irrespectively of a functional preBCR and are considered as preBCR negative^12^.

**Fig. 2:**
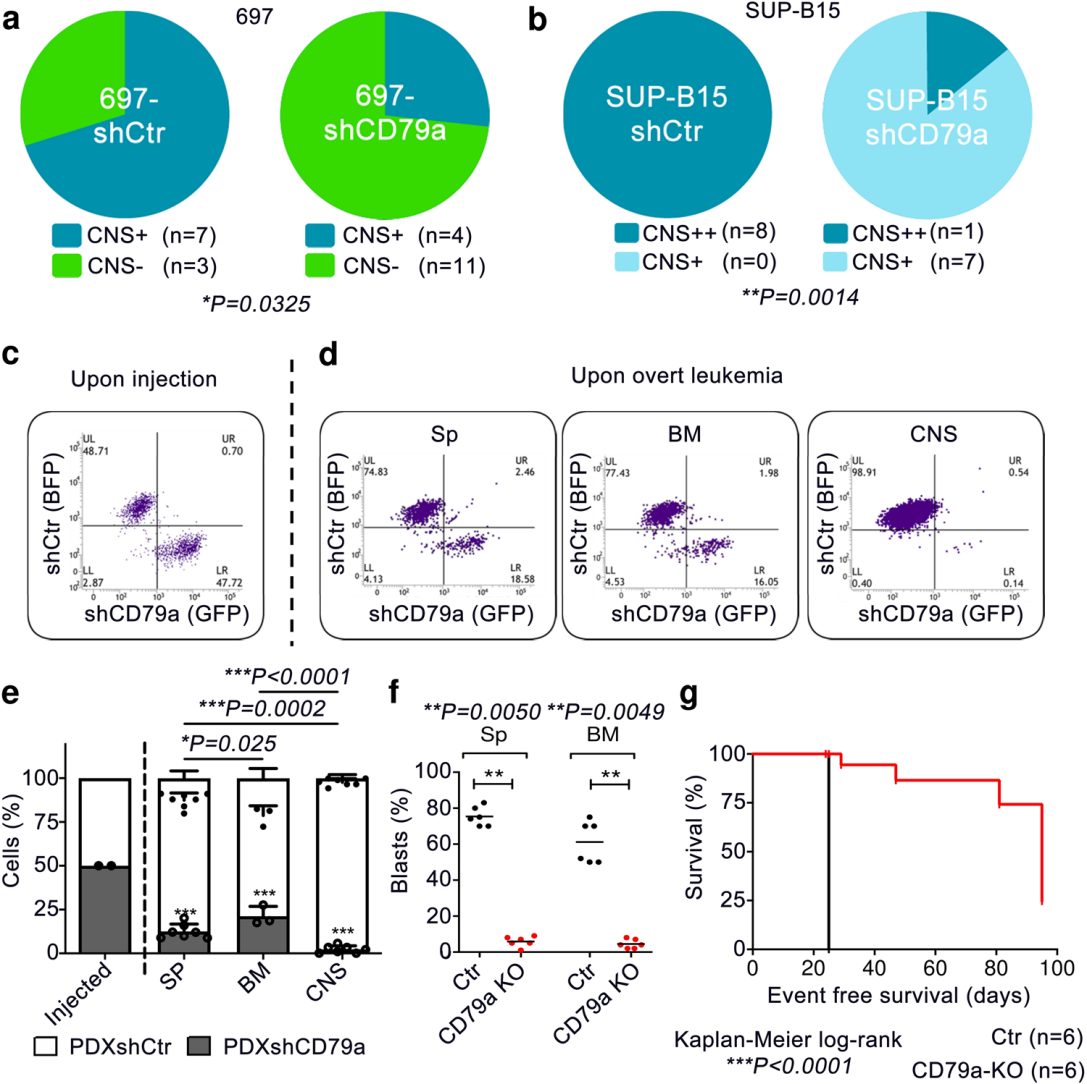
CD79a is required for leukemia engraftment and CNS-involvement in vivo. **a**, **b** One million E2A-PBX1^+^ 697 cells or BCR-ABL^+^ SUP-B15 ALL cells bearing an shRNA against CD79a (shCD79) or a control shRNA (shCtr) were injected into NSG mice and animals sacrificed when the first mouse showed signs of overt leukemia (detection of >75% leukemic blasts in the peripheral blood or clinical signs of leukemia including loss of weight or activity, organomegaly, hind-limb paralysis). Semi-quantitative scoring of CNS-involvement of **a** 697 and **b** SUP-B15 cells was employed^4^, Fisher’s exact test, two-tailed. **c**–**e** CD79a shRNA (GFP) or a control shRNA (BFP) were introduced in E2A-PBX1 positive patient cells. A total number of 2 × 10^6^ cells (1:1 ratio of both cell types) were xenografted into NSG mice (*n* = 8 animals) in a competitive experiment (**c**). Mice were sacrificed upon appearance of leukemic symptoms. **d** The count of ALL-PDX cells in spleen (Sp), bone marrow (BM), and CNS was assessed via flow cytometry and the percentages of cells bearing either shCD79 or shCtr were calculated (Sp shCtr vs. shCD79a ***P < 0.0001, BM shCtr vs. shCD79a ***P = 0.0002, CNS shCtr vs. shCD79a ***P < 0.0001) **e**, two-tailed *t*-tests, graphs show mean with standard error. **f** and **g** Mouse pro/pre-B-cells isolated from bone marrow of either wildtype (Ctr, *Mb1*^*fl/fl*^) or CD79a knockout (*Mb1*^*Cre/Cre*^, CD79a-KO) were transformed with BCR-ABL1 and injected into NSG mice (*n* = 6 Ctr animals, *n* = 12 CD79a-KO animals). *n* = 6 Ctr and *n* = 6 CD79a-KO animals were sacrificed when the control mice showed signs of overt leukemia. One group of CD79a-KO mice (*n* = 6 animals) was maintained for survival analysis. **f** percentages of leukemic cells in Sp and BM of NSG mice xenografted with Ctr or CD79a-KO cells were determined (Sp Ctr vs. CD79a KO **P = 0.0050, BM Ctr vs. CD79a KO **P = 0.0049). **g** Differences in survival of animals injected with Ctr cells (*n* = 6 animals) vs. CD79a-KO cells (*n* = 6 animals) were determined using Kaplan–Meier log-rank statistics. *P < 0.05, **P ≤ 0.01, ***P ≤ 0.001.

To further investigate this issue and the role of CD79a in CNS involvement in vivo we utilized two cell lines 697 (E2A-PBX1^+^) and SUP-B15 (BCR-ABL^+^) representing preBCR-positive and preBCR-negative BCP-ALL cells. First, we performed short-hairpin RNA-interference-mediated knockdown of CD79a in the human cell lines (Supplementary Fig. 2a). Control cells (carrying shRNA against a target sequence from *Renilla* spp., shCtr) and cells carrying CD79a shRNA (shCD79a) were injected into NSG mice and leukemia engraftment in different organs was analyzed. Indeed, animals injected with 697-shCD79a cells exposed a significant reduction of leukemic burden in the CNS (4/15 CNS^+^ animals, 26%) as compared to animals injected with control cells (7/10 CNS^+^ animals, 70%) (Fig. 2a). In contrast, leukemic infiltration in spleen and BM, as well as survival times, were comparable between both groups (Supplementary Fig. 2b–d). SUP-B15-shCD79a cells xenografted into NSG mice showed similar engraftment in the spleens and BMs as compared to SUP-B15-shCtrl cells (Supplementary Fig. 2e, f). However, animals injected with SUP-B15-shCD79a cells only showed weak infiltration of the leptomeninges whereas SUP-B15-shCtrl cells caused pronounced and multi-layered CNS-leukemia (Fig. 2b). Furthermore, animals injected with SUP-B15-shCD79a cells exposed a small but significant prolongation of median mouse survival by 12 days (Supplementary Fig. 2g), which is most likely due to the delay in development of CNS-leukemia. Together these data indicate an important role of CD79a for the engraftment of E2A-PBX1^+^ (preBCR-positive) and BCR-ABL^+^ (preBCR-negative) BCP-ALL cells in that niche.

To further substantiate the role of CD79a in CNS-involvement, CD79a knockdown experiments were performed using BCP-ALL PDX primary cells. PDX cells from an E2A-PBX1^+^ patient (Supplementary Table 1, patient 5) were stably transduced with lentiviral constructs harboring either a blue fluorescent protein (BFP) or a green fluorescent protein (GFP) reporter gene cassette. BFP cells were then stably transduced with a second vector which carried a dsRED-reporter gene coupled to the control shRNA described above (PDX-shCtr), whereas GFP cells were transduced with dsRED-reporter gene fused with a CD79a shRNA (PDX-shCD79a). Both cell types were then injected into NSG mice in a 1:1 ratio to study whether CD79a provides a niche-specific engraftment advantage (Fig. 2c, Supplementary Fig. 3a, b). Indeed, upon clinical signs of overt leukemia, significant variations in the percentage of engrafted populations of PDX-shCD79a compared to PDX-shCtr were observed in the spleen (12% vs. 88%), the BM (28% vs. 72%), and the CNS (2% vs. 98%) (Fig. 2d). Of note, the proportion of PDX-shCD79a to PDX-shCtr cells was significantly lower in PDX cells recovered from the CNS as compared to other organs (Fig. 2d, e) indicating an important role for CD79a for leukemia engraftment in mice, particularly in the CNS.

The role of CD79a in BCR-ABL^+^ ALL was further analyzed using a murine/murine transplantation model. BM cells were isolated from mice carrying either a LoxP-flanked variant of the *Mb1*-wildtype gene, which codes for CD79a (*Mb1*^*fl/fl*^; CD79a-Ctrl), or from CD79a knock-out mice (*Mb1*^*Cre/Cre*^; CD79a-KO)^13^ (Supplementary Fig. 4a). Cells were cultured for 3 days in the presence of IL7, an essential growth factor for early B-cells^14^. Subsequently, cells were transduced with a retroviral vector for BCR-ABL1 expression and cultured independent of IL7 to ensure malignant transformation^15^. CD79a-KO cells proliferated at a similar rate in vitro as compared to control (Supplementary Fig. 4b). Cells were then injected into mice. Transformed cells led to overt leukemia in mice within 29 days (exemplary BM smear in Supplementary Fig. 4c). In contrast, we found that animals injected with BCR-ABL1^+^ pro-B-cells lacking CD79a exhibited a significant delay in leukemic engraftment in the spleen, BM, and CNS (Fig. 2f, Supplementary Fig. 4d, e). No animal of the CD79a-KO group sacrificed on day 29 displayed signs of CNS-infiltration (Supplementary Fig. 4e). Importantly, animals injected with CD79a-KO cells showed a highly significant median survival prolongation of 66 days compared to mice injected with CD79a-Ctr cells (29 days vs. 95 days, Fig. 2g). These results suggest that whereas low expression levels of CD79a result in an engraftment disadvantage in the CNS, complete absence of CD79a impacts the overall engraftment of BCP-ALL cells in vivo. These findings are intriguing as previous studies have underestimated the role of preBCR signaling in BCP-ALL, particularly for subgroups which are considered preBCR negative, as they lack an assembled preBCR complex on the surface, such as BCR-ABL^+^ ALL^12^. Here we provide evidence that the signaling molecules of the preBCR are indispensable for leukemia development and CNS involvement. Supporting this view, ZAP70, PI3K, and MAPK pathways, which all act downstream the preBCR were shown to be directly involved in CNS involvement^5,16,17^. Furthermore, molecules associated with the adherence to vascular and conjunctive tissue in the meningeal microenvironment such as α6 integrin and VEGF were reported to promote CNS infiltration and survival in the CNS niche^16,18^. Hence, we assume that molecules highly abundant in the meningeal microenvironment interact with ALL cells and promote adherence and survival signaling in the CNS by crosstalk with the preBCR-signaling complex^10^. Yet, our results do not necessarily apply to the relapse situation, as we did not find increased CD79a levels in samples obtained at CNS relapse when analyzing six matched pairs of diagnostic BM samples and CNS-relapse samples in our BCP-ALL cohort. So far, small molecule inhibitors were used to regulate preBCR downstream signaling^12,16,19^. Our study promotes the view that one could also modulate this pathway by targeting the receptor itself. In this regard, IgM and CD79b antibodies, the latter being used in lymphoma treatment, may be effective options^20^. We found that not only CD79a, but also its dimerization partner CD79b is upregulated in PDX-ALL cells isolated from the CNS of xenotransplanted mice. Accordingly, prospective measurements of CD79a and CD79b in patient samples will help to validate the role of these molecules in CNS involvement in BCP-ALL.

Overall, using different pre-clinical mouse models of BCP-ALL, we show that absence of the preBCR signaling unit CD79a is associated with an attenuation of leukemic infiltration, particularly in the CNS. Hence, CD79a may represent a promising target for novel diagnosis and treatment approaches in CNS leukemia.

## Methods

### Patient samples and human cell lines

BCP-ALL patients were treated according to ALL-Berlin-Frankfurt-Münster (BFM) 2000 or 2009 protocols after informed consent in accordance with the Declaration of Helsinki. Our study was approved by the ethical committee of the Christian-Albrechts-University Kiel (D437/17). 697, SUP-B15, and REH cells were obtained from DSMZ. Authentication of cell lines was performed using flow cytometry (BCP-ALL markers such as hCD19, hCD45) and RT-PCR was used to confirm the chromosomal translocation for BCR-ABL in SUP-B15 cells. All cell lines were regularly tested for absence of mycoplasma contamination using the MycoAlert™ Mycoplasma Detection Kit (Lonza).

### BCP-ALL xenografts and isolation of PDX cells from different niches

NOD.Cg-Prkdc^scid^ Il2rg^tm1Wjl^/SzJ (NSG) mice were purchased from Charles River and xenografts generated in accordance with governmental regulations (Schleswig-Holstein Ministerium für Energiewende, Landwirtschaft, Umwelt, Natur und Digitalisierung)^4–6,21^. One million ALL cells were injected intravenously into female NSG-mice (6–10 weeks of age) and leukemic engraftment was followed by detection of human CD45^+^/murine CD45^−^/human CD19^+^ cells in the peripheral blood via flow cytometry analysis. Animals were sacrificed when showing signs of overt leukemia (detection of >75% leukemic blasts in the peripheral blood or clinical signs of leukemia including loss of weight or activity, organomegaly, hind-limb paralysis). Leukemic infiltration of the murine CNS was assessed in histological sections in blinded experiments and the scorings CNS− (no CNS infiltration of the leptomeninges), CNS+ (week infiltration of the leptomeninges) and CNS++ (strong and multilayered meningeal infiltration) were discriminated^4,6^. For the recovery of ALL cells from murine organs, mice were sacrificed by CO_2_ inhalation and spleens, hind leg bones and heads were collected. BM cells were isolated by flushing out the bones using a 27 G syringe. For extraction of the meninges, mouse skulls were opened, brains extracted and the meninges were carefully detached from the skull with tweezers. Spleen and CNS were homogenized using a 70 µm Nylon cell strainer. Spleen, BM, and CNS cells were subjected to red blood cell lysis, washed with PBS and purity of ALL cells was assessed via flow cytometry detecting human CD45, murine CD45, and human CD19.

### Flow cytometry

A minimum of 1 × 10^6^ cells were used for flow cytometry staining. Intracellular flow cytometry staining was performed using the Fixation/Permeabilization Solution Kit (BD Bioscience). Cell viability was measured using Sytox^®^ blue dead cell stain (Life Technologies). A FACS Canto II (BD Biosciences) or a MACSQuant X (Miltenyi Biotec) were used for flow cytometry. FlowJo v.10.7 was used for data analysis. Detailed information on the gating strategy is provided in Supplementary Fig. 5. Antibodies for flow cytometry (hCD19, hCD45, mCD45, and CD79a) were purchased from BioLegend. Detailed information on antibodies used for flow cytometry analysis is provided in Supplementary Table 2

### Statistics and reproducibility

Statistical analysis was performed using GraphPad PRISM 5.00, SPSS 22, SigmaPlot 12.5, and/or R v.3.3.3. Gaussian distribution was tested using the Shapiro–Wilk test. Statistical significance was assessed using an unpaired *t*-test, a Mann–Whitney test or ANOVA, depending on normal distribution and group numbers. Differences in survival were calculated using Kaplan–Meier log-rank statistics. A *P*-value of <0.05 was considered significant. The number (*n*) of independent biological replicates (at least *n* = 3) are indicated in the figure captions. Associations between gene expression and CNS status were examined by unconditional logistic regression to calculate odds ratios (ORs) and 95% CIs or using Cox proportional Hazards Model^5,22^. Bivariate correlations were analyzed calculating the Spearman correlation coefficient^5^.

### Gene expression datasets for re-evaluation

For analysis of CNS relapse in patients, the dataset from the United States National Cancer Institute “TARGET phase 1 ALL Project” was used^23^. Microarray data from diagnostic BM (*n* = 131 independent patient samples) or peripheral blood (*n* = 76 independent patient samples) of children with high risk BCP-ALL annotated with clinical follow-up data including CNS-relapse were thereby analyzed^24,25^. The dbGaP data is available via the dbGaP accession phs000218.v21.p7. The microarray data was log-transformed and *z*-scores calculated for CD79a. CD79a upregulation was defined as a *z*-score for gene expression ≥1.2 (TARGET phase 1 data set). This is equivalent to the top 11.5% CD79a-expressing patients.

### Reporting summary

Further information on research design is available in the Nature Research Reporting Summary linked to this article.

## Supplementary information

Peer Review file

Supplementary Information

Description of Additional Supplementary Files

Supplementary Data 1

Reporting Summary

## Data Availability

All data supporting the findings of this study are available within the article and its supplementary information files (Supplementary Information and Supplementary Data 1). The results published here are in part based upon data generated by the Therapeutically Applicable Research to Generate Effective Treatments (https://ocg.cancer.gov/programs/target) initiative, phs000218. The data used for this analysis are available at https://www.ncbi.nlm.nih.gov/projects/gap/cgi-bin/study.cgi?study_id=phs000218.v21.p7 (dbGaP accession: phs000218.v21.p7) managed by the United States National Cancer Institute (NCI). Microarray data used for the analysis are available on [ftp://caftpd.nci.nih.gov/pub/OCG-DCC/TARGET/ALL/clinical/Phase1/; microarray GEO accession GSE11877].
