# Supplementary material for: CD79a promotes CNS-infiltration and leukemia engraftment in pediatric B-cell precursor acute lymphoblastic leukemia
Source: Commun Biol. 2021 Jan 15;4:73. doi: 10.1038/s42003-020-01591-z (PMC7810877; doi:10.1038/s42003-020-01591-z)
Supplement: Supplementary file 2 — Supplementary Information [file 42003_2020_1591_MOESM2_ESM.pdf]

Supplementary Information

Supplementary Table 1: Clinical characteristics of BCP-ALL patients xenografted in NSG mice

Patient Data								Xenograft Data		
**Patient**	**Age (y)**	**Sex**	**WBC**	**Patient CNS[1]**	**MRD Risk[2]**	**PR[3]**	**Cytogenetic**	**% BM blasts**	**Survival (days)**	**Xenograft CNS[4] (Histology Score)**
1	14.6	M	<10.000	1	SR	G	E2A-PBX1	80, 95	94, 104	++, +
2	10.4	M	≥100.000	2a	SR	G	E2A-PBX1	97, 94	103, 79	++, ++
3	3.0	F	≥50.000 <100.000	1	SR	G	E2A-PBX1	94, N/A	97, N/A	N/A
4	8.3	F	<10.000	2b	SR	G	E2A-PBX1	100, 99	44, 55	+, ++
5 *	10.3	M	<10.000	1	IR	G	E2A-PBX1	94, 91	91, 91	++, +
6	3.8	F	≥10.000 <50.000	1	IR	G	E2A-PBX1	97, 96	102, 106	N/A
7	14.9	M	≥10.000 <50.000	1	IR	G	E2A-PBX1	97, 91	102, 84	N/A
8	3.3	F	≥10.000 <50.000	1	1	G	B-other	98, 97	280, 199	++, N/A
9	16.9	F	≥10.000 <50.000	3a	3	G	BCR-ABL1	99, 98	124, 73	++, N/A
10	5.6	M	<100.000	1	3	P	B-other	98, 90	35, 35	N/A
11	6.2	M	N/A	1	N/A	N/A	B-other	95, 99	67, 208	N/A
12	5.1	F	<10.000	3a	1	P	Hyperdiploid	100, 100	168, 153	N/A

[1]Definitions of patient CNS status in Supplementary Data.

[2]Risk stratification according to MRD risk groups: MRD-SR: TP1+2 negative, MRD-IR: TP1 and/or TP2 $<10^{-3}$, MRD-HR: TP2 $\geq 10^{-3}$.

[3]PR: Prednisone response; G: good (less than 1000 leukemic blasts/µl blood on treatment day 8); P: poor (more than 1000/µl on day 8)

[4]CNS status determined via semi quantitative scoring as published [1,2].

N/A Data not available

WBC: White blood cell count at initial diagnosis

* PDX sample used for competitive knockdown experiment

E2A-PBX1 positive patients (corresponding to Figure 1e) = Patient 1-7

Patients of other ALL cytogenetics (corresponding to Figure 1f) = Patient 8-12

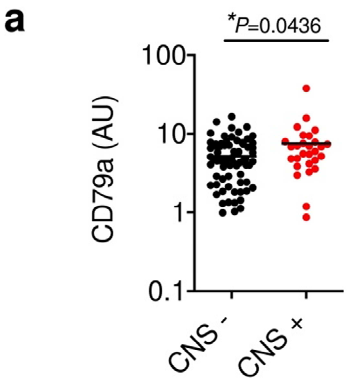

b

BCP-ALL cohort	CNS negative (n=72)		CNS positive (n=28)		Multivariate analysis		
CD79a Quartile*	No.	%	No.	%	OR†	95% CI	P-value
I	23	31.9	2	7.1	1.000§		
II, III, IV	49	68.1	26	92.9	**7.005**	1.448-33.891	**0.016**

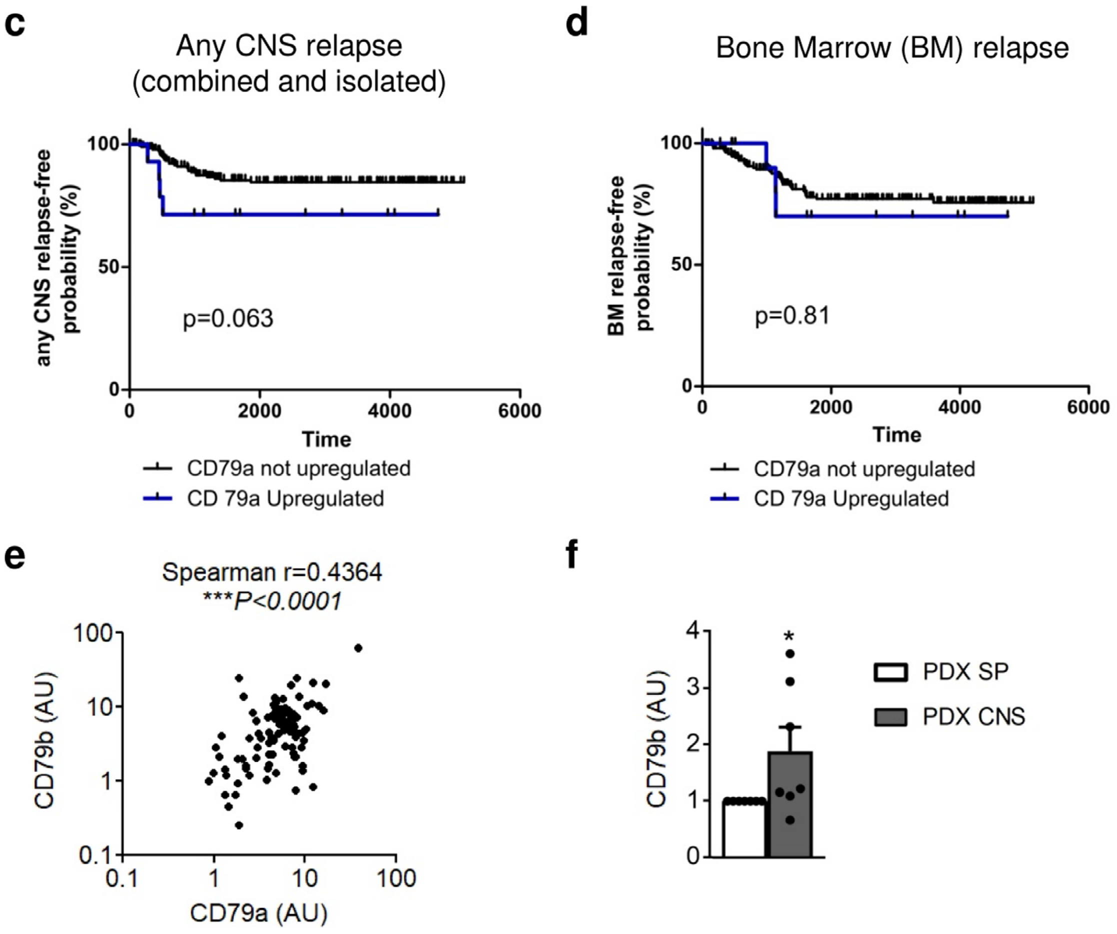

Supplementary Figure 1: CD79a is important for CNS leukemia in BCP-ALL patients and *in vivo* models (a-b) CD79a mRNA levels (normalized to mRNA levels in the 697 cell line) were measured in diagnostic BM samples in a selected cohort of 100 pediatric BCP-ALL patients of mixed cytogenetics which contained 28 CNS-positive patients matched to 72 CNS-negative patients of corresponding sex and age (including 12 TEL-AML1, 4 E2A-PBX1, 4 BCR-ABL and 3 MLL-rearranged BCP-ALL patients). (a) Expression levels of CD79a in BCP-ALL patients diagnosed as CNS-positive (CNS^+) versus CNS-negative (CNS^-) were measured, Chi-square-test, two-tailed. (b) Univariate and multivariate logistic regression analysis for risk of initial CNS-involvement, controlled for age and white blood cell (WBC) count at diagnosis as well as TEL-AML and BCR-ABL positivity. [*]Based on expression as measured by RT-PCR of patient material at initial diagnosis. [†]Multivariate OR controlled for age and WBC count at diagnosis as well as TEL-AML and BCR-ABL positivity. [§]Reference category. Definitions of patient CNS status in Supplementary Material. (c-d) Kaplan-Meier survival curve showing (c) reduced CNS-relapse-free survival rate and (d) comparable BM-relapse free survival rates in children with upregulated CD79a gene expression in diagnostic BM/peripheral blood (upregulation is defined as z-score for gene expression ≥ 1.2; TARGET phase 1 dataset). e) Bivariate correlation analysis shows a significant correlation of between CD79a and CD79b levels in diagnostic BM samples of patients of the BCP-ALL cohort of mixed cytogenetic backgrounds, Spearman correlation (two-tailed, 95% confidence interval [0.2518, 0.5903] f) E2A-PBX1+ BCP-ALL blasts from 7 different patient derived xenograft (PDX) samples were injected into NSG mice ALL cells were recovered from spleen (SP) and CNS and subjected to quantitative real time PCR (qPCR). QPCR shows the upregulation of CD79a at the transcription level in PDX cells recovered from the CNS relative to cells isolated from SP, Mann-Whitney-U test two-tailed, graphs show mean with standard error of n=7 independent samples. *=P<0.05 ***=P≤0.001 Description of xenografts is shown in Supplementary Table 1.

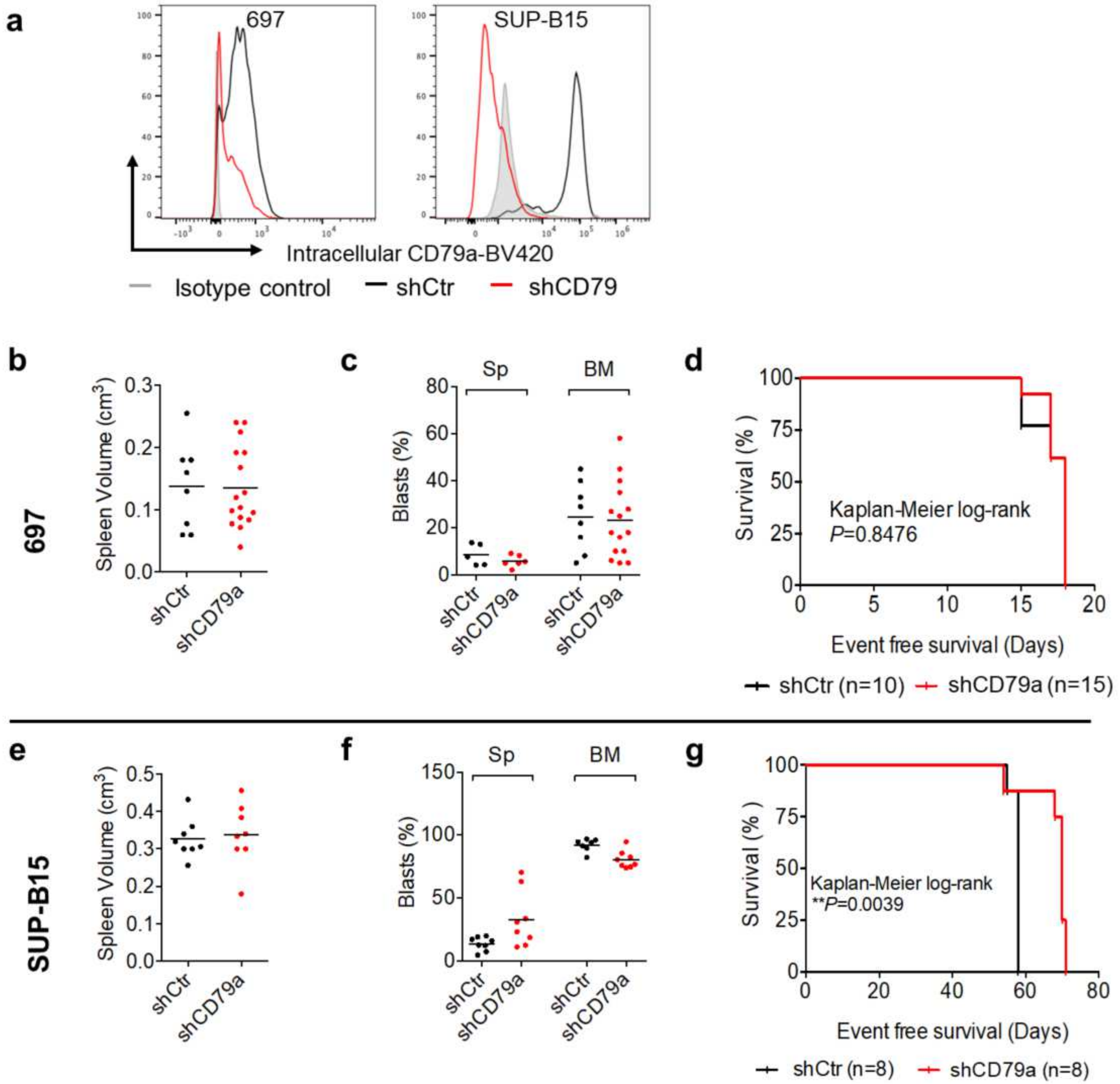

a
697
SUP-B15
Intracellular CD79a-BV420
Isotype control
shCtr
shCD79
b
697
Spleen Volume (cm³)
shCtr
shCD79a
c
Sp
BM
Blasts (%)
d
Survival (%)
Kaplan-Meier log-rank
P=0.8476
Event free survival (Days)
shCtr (n=10)
shCD79a (n=15)
e
SUP-B15
f
g
Kaplan-Meier log-rank
**P=0.0039
shCtr (n=8)
shCD79a (n=8)

Supplementary Figure 2: CD79a is required for leukemic engraftment in the CNS (a) E2A-PBX1$^+$ 697 cells or BCR-ABL$^+$ SUP-B15 ALL cells were stably transduced with an shRNA either against CD79a (shCD79a) or control (shCtr). Flow cytometry analysis was performed to validate the knockdown of CD79a. (b-d) One million E2A-PBX1$^+$ 697-shCD79a or 697-shCtr were injected into NSG mice and animals sacrificed when the first mouse showed signs of overt leukemia. One additional group was maintained for survival analysis, respectively. Animals were sacrificed either upon detection of >75% leukemic blasts in the peripheral blood or when exposing clinical leukemia (loss of weight or activity, organomegaly, hind-limb paralysis). The spleen (Sp) volume (b) and the percentage of leukemia cells in the spleen and bone marrow (BM) (c) were measured. (d) Survival curves for mice bearing either variant of 697 cells were calculated applying Kaplan-Meier log-rank statistics. (e-g) A corresponding experiment was conducted using 1 million BCR-ABL$^+$ SUP-B15 ALL cells bearing shCD79a or shCtr. The Sp volume (e) and the percentage of leukemia cells in the Sp and BM (f) were measured. (g) Survival curves for mice bearing either variant of SUP-B15 cells were calculated applying Kaplan-Meier log-rank statistics.

E2A-PBX1-PDX

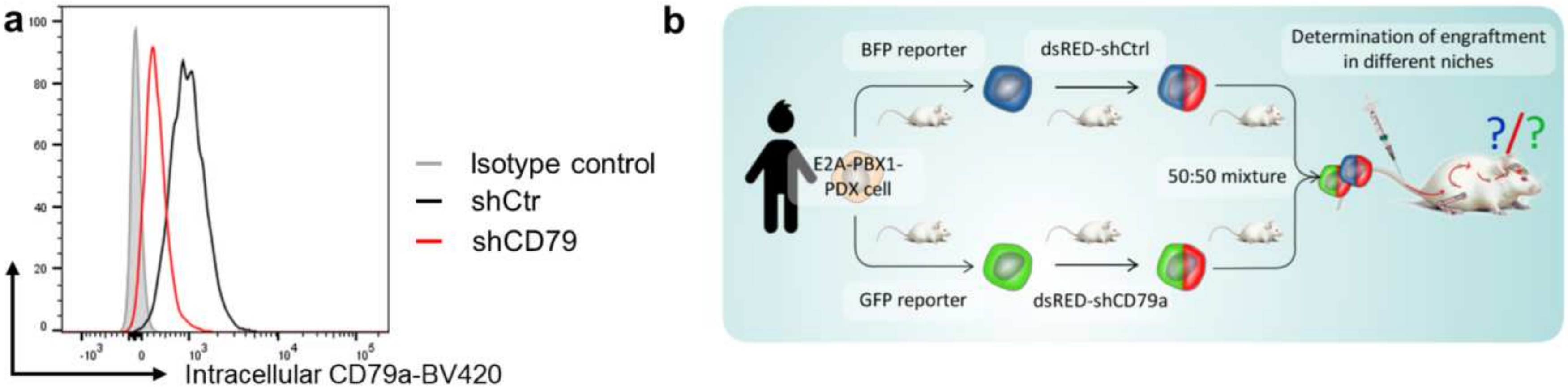

Supplementary Figure 3: Competitive CD79a-knockdown *in vivo* model CD79a shRNA (GFP) or control shRNA (BFP) were introduced into E2A-PBX1 positive patient cells. A total number of 2 x 10^6 cells (1:1 ratio of both cell types) were xenografted into NSG mice (n=8 animals) in a competitive experiment. Mice were sacrificed upon appearance of leukemic symptoms. (a) Validation of shRNA-mediated knockdown of CD79a in PDX cells as determined via flow cytometry and (b) schematic depiction of the setup of the *in vivo* experiment.

CD79a knockout in BCR-ABL+ B cell precursors

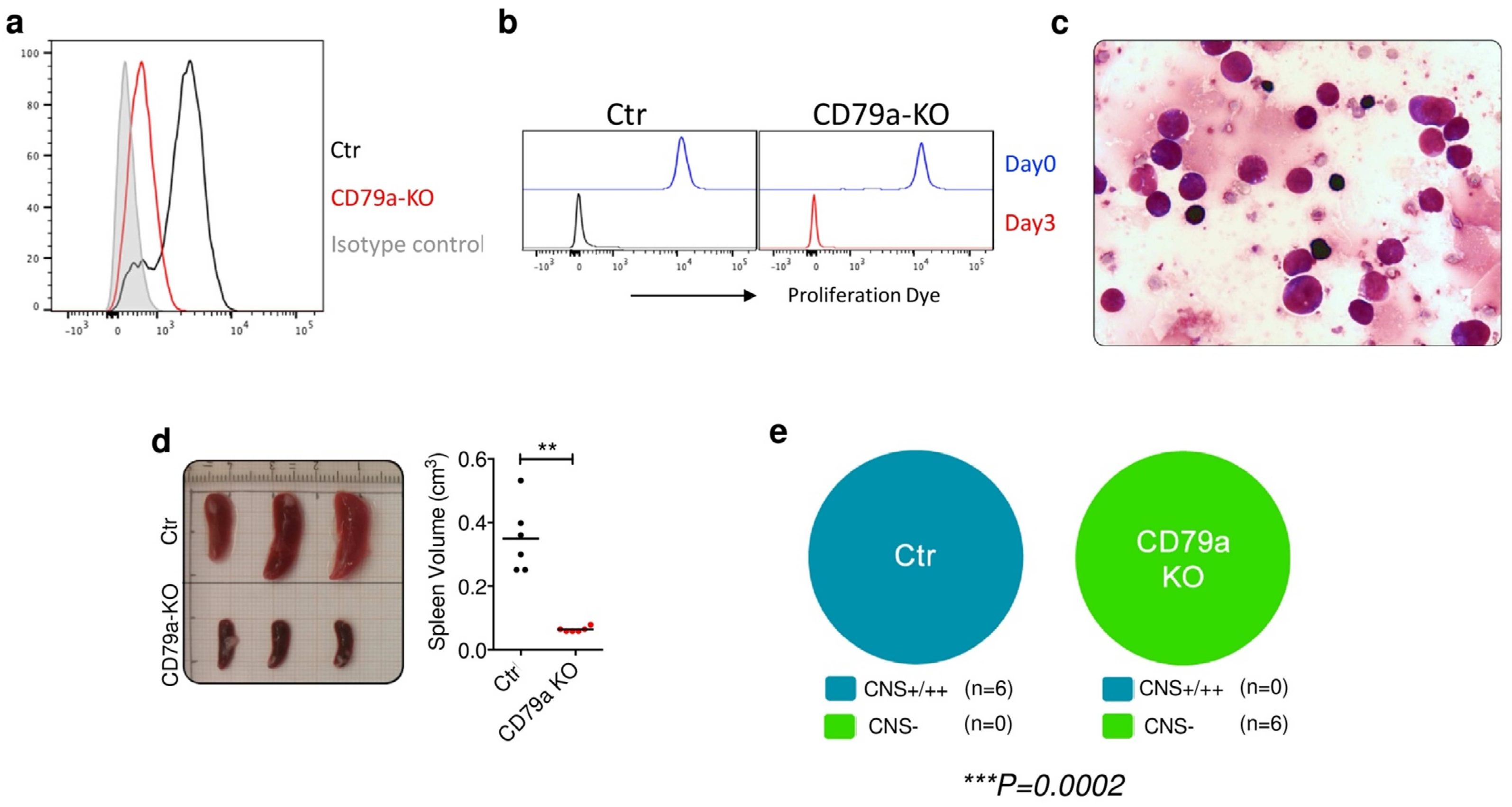

Supplementary Figure 4: CD79a is indispensable for leukemia development in BCP-ALL Mouse pro-B-cells isolated from either wildtype or CD79a knockout (*Mb1*$^{Cre/Cre}$) were transformed with BCR-ABL1. (a) Flow cytometry analysis was applied to validate the absence of CD79a. (b) To determine the *in vitro* proliferation of control cells (Ctr) or cells lacking CD79a (CD79a-KO), cells were labelled with a proliferation dye (eFluor 670) and cultured for 3 days. Cells were analyzed by flow cytometry directly after the labelling (day 0) and at the end of the experiment (day 3, one representative experiment out of three is shown). (c-e) Ctr and CD79a-KO cells were injected into NSG mice (n=6 Ctr animals, n=12 CD79a-KO animals) and n=6 CD79a-KO animals were sacrificed when the control mice showed signs of overt leukemia (day 29). One group of CD79a-KO mice (n=6 animals) was maintained for survival analysis. (c-d) Representative pictures of a (c) blood smear and (d) spleens (Sp) obtained 29 days after injection are depicted. (d) Sp volumes (unpaired t-test, two-tailed) and (e) CNS-infiltration as assessed by semi-quantitative scoring (Fisher's exact test, two-tailed) of NSG mice xenografted with Ctr or CD79a-KO cells were determined. **=$P \leq 0.01$ ***=$P \leq 0.001$

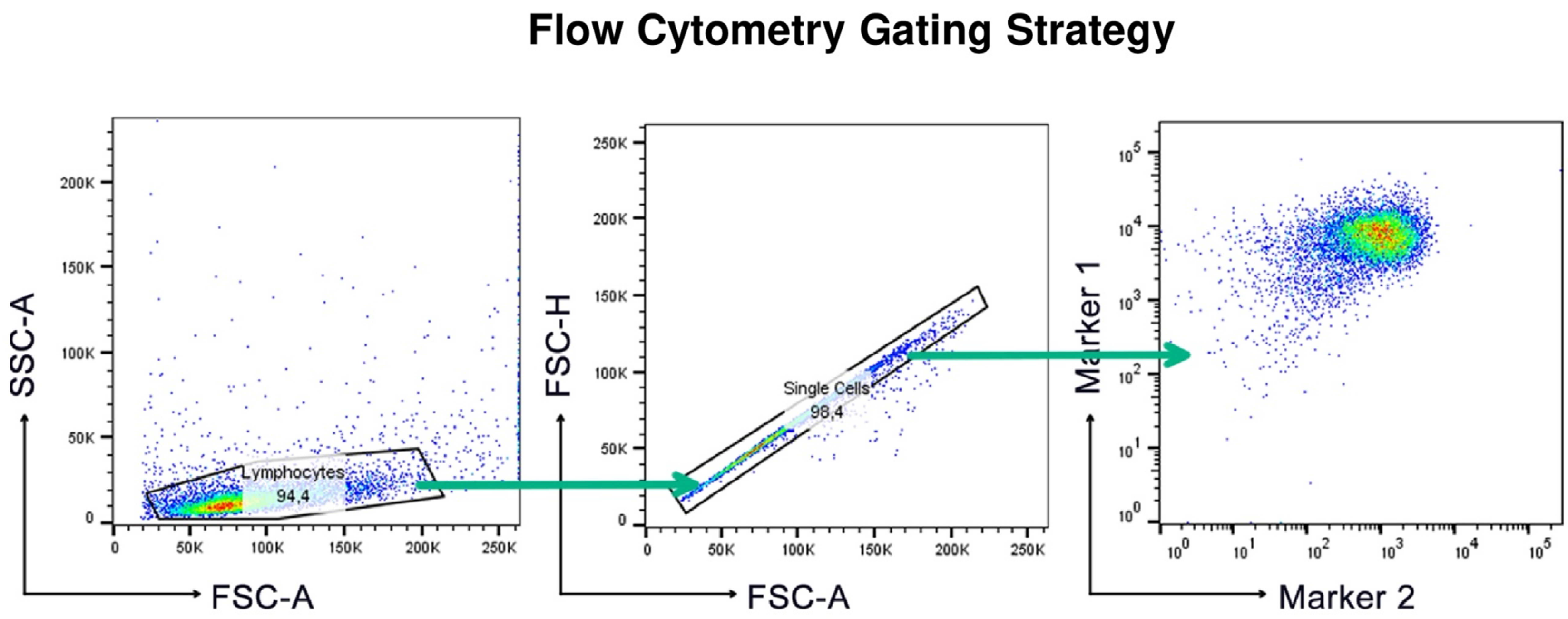

Supplementary Figure 5: Gating strategy for flow cytometry analysis: The lymphocyte gate was analyzed depending on distinguished FSC vs. SSC properties. Single cells were then selected (FSC-A vs FSC-H). The living cells were further analyzed according to surface or intracellular protein staining.

Supplementary Table 2: Antibodies used for flow cytometry

Antibody	Specificity	Host/ Isotype	Conjugate	Clone	Supplier	Catalog	Lot number	Dilution
**hCD19**	Human	Mouse/IgG1k	PE	HIB19 Monoclonal	BioLegend	302208	B273506	1:200
**mCD45**	Mouse	Rat/IgG2b	APC	I3/2.3 Monoclonal	BioLegend	147708	B237012	1:100
**hCD45**	Human	Mouse/IgG1K	FITC	HI30 Monoclonal	BioLegend	304006	B234201	1:100
**hCD79a**	Human	Mouse/IgG1K	BV421	HM47, Monoclonal	BD Horizon	562852	143568	1:20

Clinical definition of CNS status according to the ALL-BFM2009 protocol

CNS1: neither clinical nor radiological signs of CNS involvement AND no blasts in the cerebrospinal fluid (CSF) cytospin.

CNS2: neither clinical nor radiological signs of CNS involvement AND CNS2a: <10 per microliter red blood cells (RBC) and no macroscopic blood; ≤ 5 per microliter white blood cells (WBC); positive blasts in cytospin.

CNS2b: macroscopic blood and/or ≥ 10 per microliter RBC; ≤ 5 per microliter WBC; positive blasts in cytospin.

CNS2c: macroscopic blood and/or ≥ 10 per microliter RBC; >5 per microliter WBC; positive blasts in cytospin; negative according to algorithm $(WBC_L/RBC_L)/(WBC_B/RBC_B) > 2$.

CNS3-CNS3a: <10 per microliter RBC and no macroscopic blood; >5 per microliter WBC; positive blasts in cytospin.

CNS3b: macroscopic blood and/or ≥ 10 per microliter RBC; >5 per microliter WBC; positive according to algorithm $(WBC_L/RBC_L)/(WBC_B/RBC_B) > 2$.

CNS3c: clinical sings of CNS involvement, radiologically detectable cerebral lesion, retinal infiltrations.

Expression assays

Synthesis of cDNA was performed using the RevertAid First Strand cDNA Synthesis Kit (Thermo Fisher) and quantitative PCR were performed using a 7900HT Fast Real-Time PCR System (Applied Biosystems) [3]. For quantitative real time PCR, the following QIAGEN Quantitect assays were used: CD79a: #QT00014014, ZAP-70: #QT01010317, IL7R: #QT00053634 CD79b: #QT00203651, and GAPDH: #QT00079247

Knockdown experiments

Knockdown of CD79a was generated using short hairpin (sh) RNA [4]. The target sequence of the shCD79a knockdown construct (TTCATAAAGGTTTTCATCTTC) applied on BCP-ALL cell lines and PDX cells was designed with SplashRNA [5]. The shRNA sequence of the control construct applied on BCP-ALL cell lines and PDX cells (TAGATAAGCATTATAATTCCTA) is directed against a target RNA sequence from *Renilla spp.*

Establishment of murine leukemia cells

BM cells from *Mb1*$^{fl/fl}$ or *Mb1*$^{Cre/Cre}$ [6] mice were cultured for 3-7 days in Iscove's medium containing 10% heat-inactivated FCS, 2 mM L-glutamine, 100 U/ml penicillin/streptomycin, and 50µM 2-mercaptoethanol. The medium was supplemented in excess with the supernatant of J558L plasmacytoma cells stably transfected with a vector encoding murine IL7. The pro-/pre-B-cells were retrovirally transformed with an empty pMIG vector or with a pMIG vector expressing BCR-ABL1. Transformed cells were selected by IL7 withdrawal [7].

***In vitro* proliferation assay**

Cells were labelled with a proliferation dye (eFluor 670; eBioscience), cultured for 72 hours in optimum conditions [8] and measured by flow cytometry after 72 hours.

Supplementary references

1. Alsadeq, A. *et al.* IL7R is associated with CNS infiltration and relapse in pediatric B-cell precursor acute lymphoblastic leukemia. *Blood* **132,** 1614–1617; 10.1182/blood-2018-04-844209 (2018).

2. Krause, S. *et al.* Mer tyrosine kinase promotes the survival of t(1;19)-positive acute lymphoblastic leukemia (ALL) in the central nervous system (CNS). *Blood* **125,** 820–830; 10.1182/blood-2014-06-583062 (2015).

3. Alsadeq, A. *et al.* The role of ZAP70 kinase in acute lymphoblastic leukemia infiltration into the central nervous system. *Haematologica* **102,** 346–355; 10.3324/haematol.2016.147744 (2017).

4. Stief, S. M. *et al.* Loss of KDM6A confers drug resistance in acute myeloid leukemia. *Leukemia,* **34**, 50–62 1–13; 10.1038/s41375-019-0497-6 (2020).

5. Pelossof, R. *et al.* Prediction of potent shRNAs with a sequential classification algorithm. *Nature biotechnology* **35,** 350–353; 10.1038/nbt.3807 (2017).

6. Hobeika, E. *et al.* Testing gene function early in the B cell lineage in mb1-cre mice. *Proceedings of the National Academy of Sciences of the United States of America* **103,** 13789–13794; 10.1073/pnas.0605944103 (2006).

7. Hend Abdelrasoul *et al.* Synergism between IL7R and CXCR4 drives BCR-ABL induced transformation in Philadelphia chromosome-positive acute lymphoblastic leukemia. *Nat Commun* **11,** 1–12; 10.1038/s41467-020-16927-w (2020).

8. Alsadeq, A., Hobeika, E., Medgyesi, D., Kläsener, K. & Reth, M. The role of the Syk/Shp-1 kinase-phosphatase equilibrium in B cell development and signaling. *Journal of immunology (Baltimore, Md. : 1950)* **193,** 268–276; 10.4049/jimmunol.1203040 (2014).
