# Supplementary material for: CD79a promotes CNS-infiltration and leukemia engraftment in pediatric B-cell precursor acute lymphoblastic leukemia
Source: Commun Biol. 2021 Jan 15;4:73. doi: 10.1038/s42003-020-01591-z (PMC7810877; doi:10.1038/s42003-020-01591-z)
Supplement: Supplementary file 5 — Reporting Summary [file 42003_2020_1591_MOESM5_ESM.pdf]

nature research

Corresponding author(s): PD Dr. Denis Martin Schewe

Last updated by author(s): Nov 9, 2020

Reporting Summary

Nature Research wishes to improve the reproducibility of the work that we publish. This form provides structure for consistency and transparency in reporting. For further information on Nature Research policies, see our Editorial Policies and the Editorial Policy Checklist.

Statistics

For all statistical analyses, confirm that the following items are present in the figure legend, table legend, main text, or Methods section.

n/a	Confirmed	
☐	☒	The exact sample size (n) for each experimental group/condition, given as a discrete number and unit of measurement
☐	☒	A statement on whether measurements were taken from distinct samples or whether the same sample was measured repeatedly
☐	☒	The statistical test(s) used AND whether they are one- or two-sided<br>*Only common tests should be described solely by name; describe more complex techniques in the Methods section.*
☐	☒	A description of all covariates tested
☐	☒	A description of any assumptions or corrections, such as tests of normality and adjustment for multiple comparisons
☐	☒	A full description of the statistical parameters including central tendency (e.g. means) or other basic estimates (e.g. regression coefficient) AND variation (e.g. standard deviation) or associated estimates of uncertainty (e.g. confidence intervals)
☐	☒	For null hypothesis testing, the test statistic (e.g. F, t, r) with confidence intervals, effect sizes, degrees of freedom and P value noted<br>*Give P values as exact values whenever suitable.*
☒	☐	For Bayesian analysis, information on the choice of priors and Markov chain Monte Carlo settings
☒	☐	For hierarchical and complex designs, identification of the appropriate level for tests and full reporting of outcomes
☒	☐	Estimates of effect sizes (e.g. Cohen's d, Pearson's r), indicating how they were calculated

Our web collection on statistics for biologists contains articles on many of the points above.

Software and code

Policy information about availability of computer code

Data collection	No software was used for data collection
Data analysis	GraphPad PRISM 5.00, SPSS 22,68 SigmaPlot 12.5 and/or R v.3.3.3, FLowJo (v. 10.7.1)

For manuscripts utilizing custom algorithms or software that are central to the research but not yet described in published literature, software must be made available to editors and reviewers. We strongly encourage code deposition in a community repository (e.g. GitHub). See the Nature Research guidelines for submitting code & software for further information.

Data

Policy information about availability of data

All manuscripts must include a data availability statement. This statement should provide the following information, where applicable:

- Accession codes, unique identifiers, or web links for publicly available datasets
- A list of figures that have associated raw data
- A description of any restrictions on data availability

All data associated to figures or tables associated with this manuscript are available. A data availability statement is included in the manuscript.

Field-specific reporting

Please select the one below that is the best fit for your research. If you are not sure, read the appropriate sections before making your selection.

☒ Life sciences ☐ Behavioural & social sciences ☐ Ecological, evolutionary & environmental sciences

For a reference copy of the document with all sections, see nature.com/documents/nr-reporting-summary-flat.pdf

Life sciences study design

All studies must disclose on these points even when the disclosure is negative.

Sample size	No sample size calculation was applied in this study. Experiments were performed independently for at least 3 times in an unbiased manner. Animal and patient sample size was determined by experimental feasibility and sample availability to demonstrate certain results.
Data exclusions	No data was purposely omitted for the purpose of data analyses.
Replication	All in vitro studies included biological replications (three biological replicates at least) and statistics were indicated in the legends. Experiments were performed by at least two researchers to ensure reproducibility. All attempts of replication were successful. In vivo validation was conducted by an independent research team.
Randomization	Mice, with comparable weights, were randomly allocated into each treatment group.
Blinding	Investigators were blinded to group allocation and data analysis.

Reporting for specific materials, systems and methods

We require information from authors about some types of materials, experimental systems and methods used in many studies. Here, indicate whether each material, system or method listed is relevant to your study. If you are not sure if a list item applies to your research, read the appropriate section before selecting a response.

Materials & experimental systems

n/a	Involved in the study	
☐	☒	Antibodies
☐	☒	Eukaryotic cell lines
☒	☐	Palaeontology and archaeology
☐	☒	Animals and other organisms
☐	☒	Human research participants
☒	☐	Clinical data
☒	☐	Dual use research of concern

Methods

n/a	Involved in the study	
☒	☐	ChIP-seq
☐	☒	Flow cytometry
☒	☐	MRI-based neuroimaging

Antibodies

Antibodies used	Antibody, Specificity, Host/ Isotype, Conjugate, Clone, Supplier, Catalog, Lot number,Dilution<br>hCD19, Human, Mouse/IgG1k, PE, HIB19 Monoclonal, BioLegend, 302208, B273506, 1:200<br>mCD45, Mouse, Rat/IgG2b, APC, I3/2.3 Monoclonal, BioLegend, 147708, B237012, 1:100<br>hCD45, Human, Mouse/IgG1K, FITC, HI30 Monoclonal, BioLegend, 304006, B234201, 1:100<br>hCD79a, Human, Mouse/IgG1K, BV421, HM47, Monoclonal, BD Horizon, 562852, 143568, 1:20
Validation	All primary antibodies were validated commercially and reviewed by peers. Each primary antibody data provided in the manuscript has been validated for the species and application on the manufacturer's website.

Eukaryotic cell lines

Policy information about cell lines

Cell line source(s)	697 and SUPB15 cells were obtained from DSMZ
Authentication	Cell lines were authenticated using FACS (markers for BCP-ALL such as hCD19, hCD45) and RT-PCR was used to confirm the chromosomal translocation for BCR-ABL+ phenotype
Mycoplasma contamination	All cell lines were tested negative for mycoplasma contamination

Commonly misidentified lines (See ICLAC register)	No commonly misidentified lines were used in this study

Animals and other organisms

Policy information about studies involving animals; ARRIVE guidelines recommended for reporting animal research

Laboratory animals	NSG mice, female and of 8-12 weeks old.
Wild animals	No wild animals were used in this study
Field-collected samples	No field-collected samples were used in this study
Ethics oversight	NSG mice housing, breeding, and surgical procedures were approved by the governmental animal care and use committees in Schleswig-Holstein (Ministerium für Energiewende, Landwirtschaft, Umwelt, Natur und Digitalisierung).

Note that full information on the approval of the study protocol must also be provided in the manuscript.

Human research participants

Policy information about studies involving human research participants

Population characteristics	A detailed description of population characteristics is previously described in the following publication: Alsadeq et al 2018; 132, 1614–1617.
Recruitment	Patients were consecutively recruited into the clinical trials mentioned in the methods section
Ethics oversight	ALL Berlin-Frankfurt-Münster (BFM) 2000 and 2009 protocols were approved by the Ethical committee of the "Medizinische Hochschule Hannover".

Note that full information on the approval of the study protocol must also be provided in the manuscript.

Flow Cytometry

Plots

Confirm that:

☒ The axis labels state the marker and fluorochrome used (e.g. CD4-FITC).

☒ The axis scales are clearly visible. Include numbers along axes only for bottom left plot of group (a 'group' is an analysis of identical markers).

☐ All plots are contour plots with outliers or pseudocolor plots.

☐ A numerical value for number of cells or percentage (with statistics) is provided.

Methodology

Sample preparation	Samples isolated from Spleen or BM: Mice were sacrificed and specific lymphatic organs (bone marrow and spleen) were isolated and transferred into 5 ml of PBS buffer and single cell suspension was prepared. The cells were centrifuged at 1200 rpm 4°C for 5 min then resuspended in RBC lysis buffer for 3-10 min in ice. Cells were then washed with PBS, were passed through a 40 µm cell strainer and resuspended in FACS buffer. Total cell number was enumerated and dead cells were excluded by trypan blue stain. Otherwise, cells were collected directly from cell culture, washed with PBS and processed as described above.<br>Extracellular staining: 1x106 cells were transferred into a FACS tube and used for staining. The samples were centrifuged and the supernatant was removed, and the cell pellets were resuspended with 25 µl of fluorescent antibody dilution mix. The tubes were incubated on ice covered from light for 20 min. The cells were washed with 0.7-1.0 ml of FACS buffer, and the cell pellet was resuspended in 100-300 µl of FACS buffer. The tubes were kept on ice covered from light before FACS analysis (not later than 2-3 hr).<br>Intracellular staining, at least 1-2x106 cells were used. Surface staining was performed first, when needed, and then the cells were washed once with 1xPBS. Intracellular FACS staining was done using Fix and perm cell permeabilization kit (ADG) following manufacturer's instructions. After fixation 10 min at RT, cells were washed with 500 µl 1x PBS and then incubated with the primary antibody diluted in the kit-accompanied diluent for 12-20 min at RT. The cells were then washed twice with 500 µl freshly made saponin buffer (0.5% saponin, 0.5% BSA and 0.02% NaN3 in PBS). When required, labeled secondary antibody was used and the staining procedure mentioned before was repeated.
Instrument	FACS Canto II Cytometer (BD Biosciences) or MACS QuantV (Millteny Biotec) was used for flow cytometry
Software	FACSDiva (BD Biosciences) and MACSQuantify TM Software 2.11 were used for data acquiring. FlowJo v.10.1 was used for data analysis.
Cell population abundance	In general, sorting led to > 95% purity as indicated by FACS. Purity was controlled by running the post-sorted sample by FACS.
Gating strategy	Lymphocytes gate was analyzed depending on distinguished FSC vs. SSC properties. Singlets were then selected (FSC-W vs

Gating strategy	FSC-H). The living cells were further analyzed according to their surface or intracellular protein stains. At least 1x106 cells were used per staining and 100,000 total events were collected from each sample when possible. Isotype antibodies or unstained cells were used as negative controls to indicate the boundaries between negative and positive populations. The sample preparations from different organs or acquired at different timepoints were treated in a comparable way and were acquired using the same settings.

☒ Tick this box to confirm that a figure exemplifying the gating strategy is provided in the Supplementary Information.
