# Supplementary material for: CD79a promotes CNS-infiltration and leukemia engraftment in pediatric B-cell precursor acute lymphoblastic leukemia
Source: Commun Biol. 2021 Jan 15;4:73. doi: 10.1038/s42003-020-01591-z (PMC7810877; doi:10.1038/s42003-020-01591-z)
Supplement: Supplementary file 1 — Peer Review file [file 42003_2020_1591_MOESM1_ESM.pdf]

Reviewers' comments:

Reviewer #1 (Remarks to the Author):

In this study, Lennart L et al. show that increased levels of mRNA of the CD79a, a transmembrane protein and a signaling component of the preB cell receptor (preBCR), is associated with CNS-infiltration at time of diagnosis and with shorter CNS-relapse free survival in BCP-ALL pediatric cases. Furthermore, the authors show that downregulation of CD79a delay the engraftment of leukemic cells in different xenograft models. Although the data looks potentially interesting, the differences found between knocking-down of CD79a in cells lines and primary BCP-ALL leukemias, tend to suggest that CD79 could transiently affect the engraftment of BCP-ALL leukemic cells in primary and secondary mouse organs, rather than a specific role of this protein in CNS infiltration.

Specific comments/questions to the authors:

-Fig 1 (e): could you explain in material and methods how mRNA levels of CD79a in xenograft cells are corrected by endogenous (mouse) expression levels of CD79a? Are the xenograft cells purified before mRNA assessment? if it nonrequired could you explain way? Same experiment on purified hCd45/hCd19 cells would be more relevant and will give a clear answer of the expression levels of Cd79a in BCP-ALL cells in the CNS of xenografted mice.
-Assessment of the CD79a expression was measured in diagnostic BM samples in a selected cohort of 100 pediatric BCP-ALL patients of mixed cytogenetics, however the mouse models used in the paper to demonstrated the involvement of CD79a in CNS infiltration in vivo are restricted to t(1;9) and t(9,22) genetic background. It would be interesting to analyze, first, expression levels of CD79a in the different pediatric cohorts, according to the genetics (t(1;9) and t(9,22)). On the other hand, experiments with NSG mice should be carried out with a leukemia of a different genetic background.

Minor comments:
- Check probability on line 82
- Supplemental Fig. 3 a and b figure legends do not correspond to what is shown
- A detailed material and methods section could help researches to reproduce data.

Reviewer #2 (Remarks to the Author):

COMMSBIO-20-1853-T

This manuscript entitled “CD79a promotes CNS-infiltration and leukemia engraftment in pediatric B-cell precursor acute lymphoblastic leukemia” by Lenk et al shows that absence of the preBCR signaling unit CD79a is associated with decreased CNS leukemic infiltration. Although CD79a has been described previously, this manuscript offers preclinical mouse models to test the role of CD79a in CNS engraftment. The authors propose CD79a as a promising target for novel diagnosis and treatment approaches for leukemia and CNS involvement. The studies are done well, statistical analysis are appropriate, the work can be reproduced using the presented information. However, some questions remain to improve the manuscript further

1. Lines 72 and 73 and lines 90-92:

Have the authors stratified their CD79a mRNA level analysis and the CD79a and CNS-relapse analysis of TARGET phase 1 data set further by risk groups, E2A-PBX1 and BCR-ABL, which are mentioned in the introduction ? Is there an association with CD79a?

2. Line72: The authors found Cd79a expression in BM samples of diagnosis B-ALL samples. Can they comment on whether CD79a upregulation is specific for CNS relapse and less for BM relapse ? What about isolated CNS relapse ? Was CD79a expression in B-ALL compared in Diagnosis versus relapse samples ?

3. Fig. 1d:
3.1. Can the authors clarify the definition of “upregulation” and “no upregulation”, what is the percent expression cutoff of Cd79a expression ?
3.2. N=14 is small compared to n= 193 for “no upregulation”. Is there a bigger data set that authors can explore ?

4. Fig. 1e: Please clarify if Sp and BM cells enriched for human B-ALL or is there a reason why authors assume cells majority cells are B-ALL cells ?

5. Why does CD79a not impact BM engraftment but CNS infiltration ?

6. Text after line 167: Could the authors comment on CD79b, why was CD79b not included in the studies?

7. Supplementary methods: lines 50-51: The “control” needs to be clarified, is it scrambles shRNA ? The supplementary methods section does not include this information, only the main text mentions in line 107: Control cells (shCtr).

Lenk et al. 2020 Point-by-point reply to reviewer's comments

Reviewers' comments:

Reviewer #1 (Remarks to the Author): In this study, Lennart L et al. show that increased levels of mRNA of the CD79a, a transmembrane protein and a signaling component of the preB cell receptor (preBCR), is associated with CNS-infiltration at time of diagnosis and with shorter CNS-relapse free survival in BCP-ALL pediatric cases. Furthermore, the authors show that downregulation of CD79a delay the engraftment of leukemic cells in different xenograft models. Although the data looks potentially interesting, the differences found between knocking-down of CD79a in cells lines and primary BCP-ALL leukemias, tend to suggest that CD79 could transiently affect the engraftment of BCP-ALL leukemic cells in primary and secondary mouse organs, rather than a specific role of this protein in CNS infiltration.

Reply: *We thank the reviewer for the positive view of our manuscript. Based on the data shown in the manuscript, we agree with the reviewer's notion that CD79a is important not only for CNS infiltration, but also for overall leukemic engraftment in vivo. In this respect, it is important to emphasize that CD79a is mandatory for B-cell development at early (Kraus et al. 2004) as well as late stages (Torres et al. 1996). Accordingly, it was in line with our expectations that a complete deletion of CD79a as applied in our murine transplantation model (CD79aKO) had a strong effect on overall leukemic survival and CNS involvement in vivo. In the knockdown models CD79a expression was partially reduced, which gave us the opportunity to study the role of CD79a in a more differentiated manner. Our data suggest that whereas absence of CD79a prevents overall leukemia engraftment in vivo, a high CD79a expression level equips BCP-ALL cells with the ability to efficiently infiltrate or persist in the hostile CNS microenvironment. Accordingly, in all applied CD79a knockdown models, CNS engraftment is diminished and in our competitive knockdown PDX-model the downregulation of CD79a had the highest impact on BCP-ALL engraftment in the CNS as compared to other niches. We took this reviewer's comment as a motivation to further clarify our results and discuss the question why CD79a is of particular importance for CNS engraftment compared to other niches in the manuscript.*

Specific comments/questions to the authors:

Point #1: Fig 1 (e): could you explain in material and methods how mRNA levels of CD79a in xenograft cells are corrected by endogenous (mouse) expression levels of CD79a? Are the xenograft cells purified before mRNA assessment? if it nonrequired could you explain way? Same experiment on purified hCd45/hCd19 cells would be more relevant and will give a clear answer of the expression levels of Cd79a in BCP-ALL cells in the CNS of xenografted mice.

Reply: *We consent with the reviewer that murine B-cells also express high levels of endogenous CD79a. However, the CD79a expression measured here is specific to ALL cells based on the nature of the applied xenograft model: NSG-mice are completely lacking a B-cell and T-cell compartment (Shultz et al. 2007). As CD79a is highly specific to B-cells, CD79a expression endogenous to murine cells can be excluded. Moreover, upon full leukemic engraftment, BCP-ALL-PDX cells make up 95-100% of the cell population in our NSG-mouse model in the relevant organs. Red blood cells are lysed upon sample*

*preparation. Hence, CD79a levels measured here can reliably be assigned to engrafted PDX cells of human origin. For clarification, we include a representative flow cytometry analysis in this letter, which shows the population of leukemia cells after isolation from the respective niche (**Figure 1**). We think that this type of analysis is not interesting enough to show it in the manuscript. Furthermore, we added information on the isolation of PDX-ALL cells from different niches to the methods section as requested by the reviewer.*

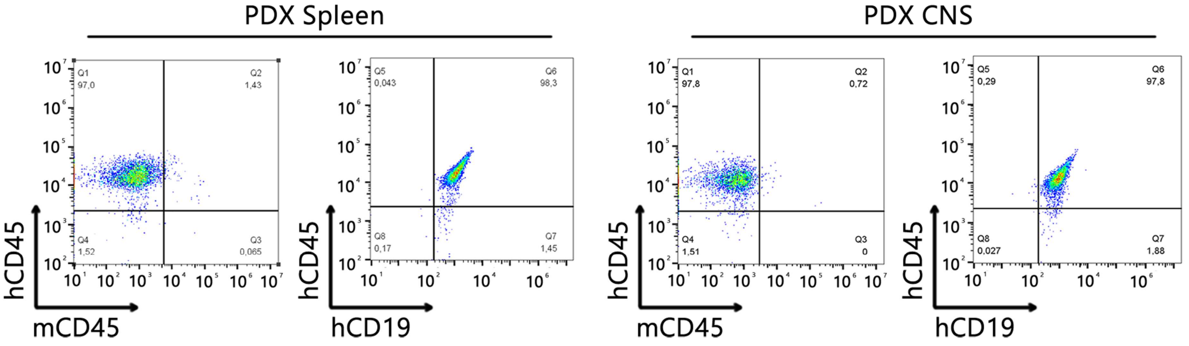

Figure 1: *Representative flow cytometric characterization of cells isolated from the murine spleen and CNS via staining of human-CD45, murine CD45 and human CD19. The data show high purity of BCP-ALL- patient derived xenograft (PDX) cells after isolation from NSG mouse organs.*

Point#2: Assessment of the CD79a expression was measured in diagnostic BM samples in a selected cohort of 100 pediatric BCP-ALL patients of mixed cytogenetics, however the mouse models used in the paper to demonstrated the involvement of CD79a in CNS infiltration in vivo are restricted to t(1;9) and t(9,22) genetic background. It would be interesting to analyze, first, expression levels of CD79a in the different pediatric cohorts, according to the genetics (t(1;9) and t(9,22)). On the other hand, experiments with NSG mice should be carried out with a leukemia of a different genetic background.

Reply:

2.1 *We thank the reviewer for this important remark. Indeed, based on our cohort of mixed cytogenetics (including 12 TEL-AML1, 4 E2A-PBX1, 4 BCR-ABL and 3 MLL-rearranged BCP-ALL patients), we claim that CD79a is important for CNS infiltration irrespective of cytogenetics. This finding was intriguing to us as previous reports divided different cytogenetic leukemia subtypes into such that critically depend on preBCR-signaling (e.g. E2A-PBX1 positive BCP-ALL) versus those that progress independently of preBCR signaling (e.g. BCR-ABL positive leukemia) (Geng et al. 2015). As CD79a is expressed in all cytogenetic subtypes (**Figure 2**), we hypothesized that this molecule may be important for CNS infiltration irrespective of a functionally assembled preBCR signaling complex (which is composed of the signaling molecules CD79a/b and µHC and the surrogate light chain). Accordingly, we chose E2A-PBX1 positive leukemia cells as representative of a cytogenetic subtype commonly referred to as preBCR positive (Geng et al. 2015) and BCR-ABL positive leukemia cells representing preBCR negative-BCP-ALL. We clarified this critical point in the text of the manuscript.*

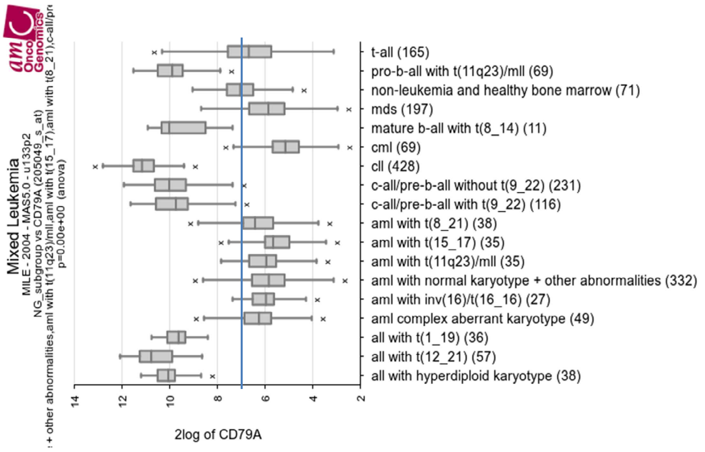

Figure 2: *Expression levels of CD79a in different mixed leukemia dataset as extracted from the Microarray Innovations in LEukemia (MILE) study 2004 dataset (Kohlmann et al. 2008). The blue line represents the median expression levels of CD79a in healthy samples.*

Nevertheless, the fact that E2A-PBX1 and BCR-ABL positive leukemia are considered CNS-tropic, leads to the suggestion that high CD79a levels should be associated with CNS status in these subgroups. Based on the reviewer suggestion, we further investigated the association of CNS involvement with respect to patient cytogenetics. Therefore, we measured CD79a levels in a cohort of 68 BCR-ABL positive patients (5 CNS-positive patients) that we had published previously (Abdelrasoul et al. 2020). We found no significant differences in CD79a levels in CNS+ versus CNS- patients in the cohort of BCR-ABL positive patients. Furthermore, we measured CD79a mRNA levels in a previously published unselected cohort of 61 E2A-PBX1 patients (6 CNS-positive patients (Krause et al. 2015). We found slightly higher mRNA levels of CD79a in CNS-positive patients compared to patients diagnosed as CNS-negative which did not reach statistical significance. However, the overall low number of CNS positive patients in the investigated cohorts did not contain enough CNS-positive patients to draw clear conclusions concerning the role of CD79a in CNS leukemia in these subgroups. As the two BCP-ALL cohorts utilized in our study contain a high number of patients diagnosed CNS+, these cohorts are particularly eligible for analyses concerning CNS leukemia.

2.2 *To further address the issue raised by the reviewer, we measured CD79a levels in PDX cells isolated from the CNS and spleen of NSG-mice injected with bone marrow samples from BCP-ALL patients of further cytogenetic leukemia subtypes (1xBCR-ABL, 1xHyperdiploid, 3x "B-other") and the highly CNS tropic cell line REH (TEL-AML1). Indeed, in 5 out of 6 ALL-transplanted mice, we found higher CD79a levels in ALL cells isolated from the CNS compared to spleen indicating a role of CD79a in CNS involvement in further cytogenetic BCP-ALL subtypes. We added the corresponding panel to the results section (**Figure 1f**).*

Minor comments:

Point 1#: - Check probability on line 82

Reply: *We thank the reviewer for pointing out this mistake. The denoted p-value has been corrected in the revised manuscript.*

Point #2: - Supplemental Fig. 3 a and b figure legends do not correspond to what is shown.

Reply: *We apologize for the error and the labelling in the figure legend has been changed accordingly.*

Point #3: - A detailed material and methods section could help researches to reproduce data.

Reply: *To match the journal guidelines we had included the methods section into the supplementary part of the manuscript. In accordance with the reviewer suggestion, we added further methodological details into the methods section to help other researchers to reproduce the data.*

Reviewer #2 (Remarks to the Author): This manuscript entitled "CD79a promotes CNS-infiltration and leukemia engraftment in pediatric B-cell precursor acute lymphoblastic leukemia" by Lenk et al shows that absence of the preBCR signaling unit CD79a is associated with decreased CNS leukemic infiltration. Although CD79a has been described previously, this manuscript offers preclinical mouse models to test the role of CD79a in CNS engraftment. The authors propose CD79a as a promising target for novel diagnosis and treatment approaches for leukemia and CNS involvement. The studies are done well, statistical analysis are appropriate, the work can be reproduced using the presented information. However, some questions remain to improve the manuscript further.

Reply: *We thank the reviewer for the positive comments.*

Point #1: Lines 72 and 73 and lines 90-92: Have the authors stratified their CD79a mRNA level analysis and the CD79a and CNS-relapse analysis of TARGET phase 1 data set further by risk groups, E2A-PBX1 and BCR-ABL, which are mentioned in the introduction? Is there an association with CD79a?

Reply: *We thank the reviewer for this interesting question. Indeed, it would be of great interest to find out if CD79a expression is associated with CNS infiltration and relapse in particular subgroups of BCP-ALL to increase diagnostic accuracy and predict the probability of CNS relapse. To address this question we first performed further analysis with the TARGET cohort. Unfortunately, patients expressing the BCR-ABL fusion gene were not included into the study so that no further information could be gained in this respect. The cohort contained 23 E2A-PBX1 positive patients. We did not find an association of CD79a mRNA levels with risk for isolated CNS relapse.*

*As stated above (**Reply 2.1** to reviewer 1), to further investigate the association of CNS involvement with respect to patient cytogenetics, we measured CD79a levels in a cohort of 68 BCR-ABL positive patients (5 CNS-positive patients) that we had published previously (Abdelrasoul et al. 2020). We found no significant differences in CD79a levels in CNS+ versus CNS- patients in the cohort of BCR-ABL positive patients. Furthermore, we measured CD79a mRNA levels in a previously published unselected cohort of 61 E2A-PBX1 patients (6 CNS-positive patients (Krause et al. 2015)). We found slightly higher mRNA levels of CD79a in CNS-positive patients compared to patients diagnosed as CNS-negative which did not reach statistical significance. However, due to the overall low number of CNS positive patients in these cohorts clear conclusions concerning the role of CD79a in CNS leukemia and CNS relapse in these subgroups could not be drawn. Yet, the newly generated data from NSG-mice bearing BCP-ALL-PDX cells from other entities show higher CD79a mRNA levels*

in BCP-ALL cells isolated from the CNS compared to spleen (new ***Figure 1f*** *in the main manuscript, reply 2.2 to reviewer 1). This further supports the view that the role of CD79a in CNS involvement applies to BCP-ALL in general, irrespective of cytogenetics.*

Point #2: Line72: The authors found Cd79a expression in BM samples of diagnosis B-ALL samples. Can they comment on whether CD79a upregulation is specific for CNS relapse and less for BM relapse ? What about isolated CNS relapse ? Was CD79a expression in B-ALL compared in Diagnosis versus relapse samples ?

Reply: ***2.1*** *We thank the reviewer for giving us the opportunity to clarify this issue: In our initial analysis of the TARGET cohort, we only analyzed the risk for* ***isolated CNS relapse****. Inspired by the reviewer comments, we re-analyzed the TARGET dataset and we found a tendency towards an increased risk for any kind of CNS relapse in patients with high CD79a levels. Interestingly, we found no association between CD79a levels and bone marrow relapse further promoting the view that CD79a is important for relapse events linked to the CNS. We added the corresponding figures into* ***Supplementary Fig. 1c-d****.*

2.2. *To investigate whether CD79a expression varies in B-ALL samples obtained at diagnosis versus relapse, we measured CD79a expression in 6 matched-pair samples obtained from initial diagnosis versus CNS relapse of patients from our BCP-ALL cohort of mixed cytogenetics. We did not find a statistically significant difference between samples from diagnosis versus CNS relapse. Nevertheless, we added this information into the main text of the manuscript. Interestingly, the CNS relapsed samples showed slightly but not significantly lower CD79a levels relative to samples obtained at initial diagnosis. These preliminary data suggest that although an increased CD79a expression facilitates CNS involvement at initial diagnosis and may predict relapse, ALL cells may tend to downregulate CD79 at later stages of the disease for example at relapse. CD79a, as part of pre-BCR signaling, is thought to be responsible in mediating survival as well as proliferation signals. It is possible that a slight downregulation of CD79a could be required to modulate signaling thresholds in the CNS microenvironment and may provide a coping mechanism for ALL cells to acquire long-term survival advantages in that niche.*

At this point, based on our data, we can only state that high levels of CD79a upon diagnosis are associated with a higher risk for relapse events associated with the CNS. The question why CD79a is particularly important for CNS involvement and CNS relapse is a major subject of ongoing research in our group. At this point, we hypothesize that the high abundance of certain ligands in the CNS shown to stimulate pathways associated with the preBCR (e. g. Laminins and Galectins) may particularly promote survival signaling in BCP-ALL cells helping them to adapt to and colonize the hostile CNS microenvironment (reviewed in Lenk et al. 2020a and Lenk et al. 2020b). We therefore included these hypotheses into the discussion.

Point #3.1: Fig. 1d: Can the authors clarify the definition of “upregulation” and “no upregulation”, what is the percent expression cutoff of Cd79a expression ?

Reply: *We thank the reviewer for this clarifying hint. Our analysis depicts a Kaplan-Meier survival curve showing reduced isolated CNS (iCNS) relapse-free probability in children with upregulated CD79a gene expression in diagnostic BM (n = 131) or peripheral blood (n = 76) samples of children with high-risk ALL. CD79a upregulation was defined as a z score for gene expression ≥1.2 (TARGET phase 1 data set). This is equivalent to the 11.5% top*

CD79a-expressing patients. We included this information into the methods section for clarification and the Legends of Figure 1 and Supplementary Figure 1.

Point #3.2: N=14 is small compared to n= 193 for "no upregulation". Is there a bigger data set that authors can explore ?

Reply: *We agree with this reviewer that the group size of patients with CD79a upregulation is limited and that a bigger dataset could help. This is actually the case with any kind of analysis regarding CNS-positive ALL patients. We carefully checked the databases and existing literature for further datasets to extend our data. Unfortunately, to our knowledge no datasets bigger than the ones at hand are available to investigate CNS involvement. More recent versions of the TARGET database (phase II) contain lower numbers of patients with CNS-associated events. In an ongoing study, we are prospectively measuring CD79a levels in a large unselected cohort of BCP-ALL patients in the AIEOP-BFM 2017 study, so that we hope to contribute to this issue in the future. Nevertheless, we have addressed these points in the discussion of the revised manuscript.*

Point # 4: Fig. 1e: Please clarify if Sp and BM cells enriched for human B-ALL or is there a reason why authors assume cells majority cells are B-ALL cells ?

Reply: *Please see response to reviewer 1 point #1)*

Point # 5: Why does CD79a not impact BM engraftment but CNS infiltration ?

Reply: *As stated above (response 2.2 to reviewer 1), our data show that CD79a is important for overall leukemic engraftment in vivo in different niches including the bone marrow. However, based on our data generated in our CD79-knockdown models we think that the unique microenvironment of the CNS provides BCP-ALL cells highly expressing CD79a with factors that promote their ability to infiltrate and colonize the CNS niche. Various pathways associated with adherence and survival in the CNS niche have recently been described (reviewed in Lenk et al. 2020a and Lenk et al. 2020b). We hypothesize that the preBCR molecules CD79a and CD79b are particularly important for the communication of the CNS niche with these pathways in BCP-ALL cells. We added some concluding remarks and discussion sentences in this regard into the main text of the manuscript.*

Point #6: Text after line 167: Could the authors comment on CD79b, why was CD79b not included in the studies?

Reply: *In line with previous reports stating that CD79a and CD79b form an obligatory heterodimer Müschen. 2015 we like to state that our current research indicates that CD79b also plays a role in leukemia development and CNS infiltration. In this regard, we added further results to the manuscript showing a strong positive correlation between CD79a and CD79b levels in diagnostic bone marrow samples in our BCP-ALL patient cohort. Moreover, we found that like CD79a, CD79b is upregulated in E2A-PBX1 positive PDX cells isolated from the CNS of NSG-mice compared to PDX cells recovered from the bone marrow further underpinning our hypothesis. We added these data to the supplementary section of the*

*manuscript (**Supplementary Figure 1e-f**). The role of CD79b in B-cell leukemia is an ongoing research topic in our group, and we are confident to be able to show further evidence in an independent piece of work in the near future.*

Point #7: Supplementary methods: lines 50-51: The “control” needs to be clarified, is it scrambles shRNA ? The supplementary methods section does not include this information, only the main text mentions in line 107: Control cells (shCtr).

Reply:

We thank the authors for this clarifying suggestion. The shRNA sequence of the control construct is a 22-mer directed against a protein from Renilla spp. Both, the mentioned shCD79a sequence and the shCtrl target sequences were applied in the knockdown experiments with BCP-ALL cell lines as well as in PDX cells. This information and the control shRNA-sequence (TAGATAAGCATTATAATTCCTA) have been added to the methods section.

References

1. Kraus *et al.* Survival of resting mature B lymphocytes depends on BCR signaling via the Igalpha/beta heterodimer. *Cell* **117,** 787–800; 10.1016/j.cell.2004.05.014 (2004).

2. Torres *et al.* Aberrant B cell development and immune response in mice with a compromised BCR complex. *Science (New York, N.Y.)* **272,** 1804–1808; 10.1126/science.272.5269.1804 (1996).

3. Shultz *et al.* Humanized mice in translational biomedical research. *Nature reviews. Immunology* **7,** 118–130; 10.1038/nri2017 (2007).

4. Geng *et al.* Self-enforcing feedback activation between BCL6 and pre-B cell receptor signaling defines a distinct subtype of acute lymphoblastic leukemia. *Cancer cell* **27,** 409–425; 10.1016/j.ccell.2015.02.003 (2015).

5. Kohlmann *et al.* An international standardization programme towards the application of gene expression profiling in routine leukaemia diagnostics. The Microarray Innovations in LEukemia study prephase. *British journal of haematology* **142,** 802–807; 10.1111/j.1365-2141.2008.07261.x (2008).

6. Hend Abdelrasoul *et al.* Synergism between IL7R and CXCR4 drives BCR-ABL induced transformation in Philadelphia chromosome-positive acute lymphoblastic leukemia. *Nat Commun* **11,** 1–12; 10.1038/s41467-020-16927-w (2020).

7. Krause *et al.* Mer tyrosine kinase promotes the survival of t(1;19)-positive acute lymphoblastic leukemia (ALL) in the central nervous system (CNS). *Blood* **125,** 820–830; 10.1182/blood-2014-06-583062 (2015).

8. Lenk *et al.* Involvement of the central nervous system in acute lymphoblastic leukemia. Opinions on molecular mechanisms and clinical implications based on recent data. *Cancer metastasis reviews*, **39**, 173–187 10.1007/s10555-020-09848-z (2020a).

9. Lenk *et al.* When the bond breaks - targeting adhesion of leukemia cells to the meninges. *Haematologica* **105,** 1991–1993; 10.3324/haematol.2020.253609 (2020b).

10. Müschen. Rationale for targeting the pre-B-cell receptor signaling pathway in acute lymphoblastic leukemia. *Blood* **125,** 3688–3693; 10.1182/blood-2015-01-567842 (2015).

REVIEWERS' COMMENTS:

Reviewer #1 (Remarks to the Author):

Thank you for your comments, clarifications and additional information.

All my concerns have been responded and assessed. However, I still I am a bit surprised about the efficacy of the BCP-ALL leukemias engraftment in the CNS of NSG mice. According supplementary table 1 and, although that the number of leukemias assessed is low and the CNS status data missed for some leukemias, leukemic cells are found in the meninges of the NSG mice even when primary sample does not present CNS infiltration in the patient (score 1, 2a ALL-BFM2009), do you know why?

Reviewer #2 (Remarks to the Author):

All comments were addressed well and the manuscript has improved significantly. The work is now convincing and I have no further comments.

Lenk *et al.* 2020 - Nature Communications Biology - Point-by-point reply to reviewer's comments

Reviewer #1 comment: All my concerns have been responded and assessed. However, I still I am a bit surprised about the efficacy of the BCP-ALL leukemias engraftment in the CNS of NSG mice. According supplementary table 1 and, although that the number of leukemias assessed is low and the CNS status data missed for some leukemias, leukemic cells are found in the meninges of the NSG mice even when primary sample does not present CNS infiltration in the patient (score 1, 2a ALL-BFM2009), do you know why?

Response: We thank the reviewer for the affirmative review of our manuscript and for raising these interesting remarks. Indeed, we found some degree of meningeal infiltration in all examined patient derived xenograft (PDX) animals irrespective of the CNS status of the corresponding ALL patient. This finding is in accordance with a previous report from Williams *et al.* that found CNS engraftment in 23 of 29 NSG-mice xenotransplanted with ALL cells from different patients, of which 21 were CNS-negative by lumbar puncture (Williams et al., 2016). Also, our own data (Krause et al., 2015; Alsadeq et al., 2017; Alsadeq et al., 2018) show that most BCP-ALL patients usually show some degree of CNS infiltration in xenotransplanted mice. These findings allow the conclusion that BCP-ALL cells are in principle able to infiltrate the CNS, further promoting the clinical view that patients with ALL are probably almost always CNS positive, but below the detection limits of the assays currently used as standard diagnostics in the clinic. This also matches old autopsy studies confirming high numbers of CNS positive patients (Price and Johnson, 1973), which is what we see in mice. The degree of CNS engraftment in mice may still also dependent on patient features, e. g. the genetic makeup of the cells, the round of xenografting (primografts vs. secondary or tertiary transplantations) and murine factors such as the degree of immunodeficiency (NSG vs. NOD-SCID mice). Also, from our own data (Krause et al., 2015), we can infer that CNS positive ALL patients usually show a high degree of CNS infiltration in mice when a semi-quantitative scoring method is applied and that semi-quantitative scoring is a valid method to assess the efficacy of a specific therapy on CNS infiltration. It is also possible that the infiltration routes of ALL cells into the CNS vary between species (reviewed in Lenk et al., 2020), so that there may be inherent limitations in this type of xenograft models. We included a clarifying sentence referring to this comment in the manuscript (line 132).

Reviewer #2 comment: All comments were addressed well and the manuscript has improved significantly. The work is now convincing and I have no further comments.

Response: We thank the reviewer for the positive view of our manuscript and the previous helpful comments.

References

Alsadeq, A., Fedders, H., Vokuhl, C., Belau, N.M., Zimmermann, M., Wirbelauer, T., Spielberg, S., Vossen-Gajcy, M., Cario, G., and Schrappe, M., et al. (2017). The role of ZAP70 kinase in acute lymphoblastic leukemia infiltration into the central nervous system. Haematologica *102*, 346-355.

Alsadeq, A., Lenk, L., Vadakumchery, A., Cousins, A., Vokuhl, C., Khadour, A., Vogiatzi, F., Seyfried, F., Meyer, L.-H., and Cario, G., et al. (2018). IL7R is associated with CNS infiltration and relapse in pediatric B-cell precursor acute lymphoblastic leukemia. Blood *132*, 1614-1617.

Krause, S., Pfeiffer, C., Strube, S., Alsadeq, A., Fedders, H., Vokuhl, C., Loges, S., Waizenegger, J., Ben-Batalla, I., and Cario, G., et al. (2015). Mer tyrosine kinase promotes the survival of t(1;19)-positive acute lymphoblastic leukemia (ALL) in the central nervous system (CNS). Blood *125*, 820-830.

Lenk, L., Alsadeq, A., and Schewe, D.M. (2020). Involvement of the central nervous system in acute lymphoblastic leukemia. Opinions on molecular mechanisms and clinical implications based on recent data. Cancer metastasis reviews.

Price, R.A., and Johnson, W.W. (1973). The central nervous system in childhood leukemia. I. The arachnoid. Cancer *31*.

Williams, M.T.S., Yousafzai, Y.M., Elder, A., Rehe, K., Bomken, S., Frishman-Levy, L., Tavor, S., Sinclair, P., Dormon, K., and Masic, D., et al. (2016). The ability to cross the blood-cerebrospinal fluid barrier is a generic property of acute lymphoblastic leukemia blasts. Blood *127*, 1998-2006.
